# Vitamin A Affects Flatfish Development in a Thyroid Hormone Signaling and Metamorphic Stage Dependent Manner

**DOI:** 10.3389/fphys.2017.00458

**Published:** 2017-06-30

**Authors:** Ignacio Fernández, Juan B. Ortiz-Delgado, Maria J. Darias, Francisco Hontoria, Karl B. Andree, Manuel Manchado, Carmen Sarasquete, Enric Gisbert

**Affiliations:** ^1^Centro de Ciências do Mar (CCMAR), Universidade do Algarve Faro, Portugal; ^2^Instituto de Ciencias Marinas de Andalucía (CSIC) Cádiz, Spain; ^3^Unité Mixte de Recherche Biologie des Organismes et Ecosystèmes Aquatiques, Institut de Recherche Pour le Développement Montpellier, France; ^4^Instituto de Acuicultura de Torre de la Sal (CSIC) Torre de la Sal, Castellón, Spain; ^5^Unitat de Cultius Experimentals, Centre de Sant Carles de la Ràpita, Institute for Research and Technology in Food and Agriculture Sant Carles de la Ràpita, Spain; ^6^IFAPA Centro “El Toruño,” Junta de Andalucía, El Puerto de Santa Maria Cádiz, Spain

**Keywords:** vitamin A, thyroidal follicles, skeletogenesis, ossification, nuclear receptors, flatfish, Senegalese sole, *Solea senegalensis*

## Abstract

Vitamin A (VA) and retinoid derivatives are known morphogens controlling vertebrate development. Despite the research effort conducted during the last decade, the precise mechanism of how VA induces post-natal bone changes, and particularly those operating through crosstalk with the thyroid hormones (THs) remain to be fully understood. Since effects and mechanisms seem to be dose and time-dependent, flatfish are an interesting study model as they undergo a characteristic process of metamorphosis driven by THs that can be followed by external appearance. Here, we studied the effects of VA imbalance that might determine Senegalese sole (*Solea senegalensis*) skeletogenetic phenotype through development of thyroid follicles, THs homeostasis and signaling when a dietary VA excess was specifically provided during pre-, pro- or post-metamorphic stages using enriched rotifers and *Artemia* as carriers. The increased VA content in enriched live prey was associated to a higher VA content in fish at all developmental stages. Dietary VA content clearly affected thyroid follicle development, T3 and T4 immunoreactive staining, skeletogenesis and mineralization in a dose and time-dependent fashion. Gene expression analysis showed that VA levels modified the mRNA abundance of VA- and TH-specific nuclear receptors at specific developmental stages. Present results provide new and key knowledge to better understand how VA and TH pathways interact at tissue, cellular and nuclear level at different developmental periods in Senegalese sole, unveiling how dietary modulation might determine juvenile phenotype and physiology.

## Introduction

Skeletogenesis is a key morphogenetic event in the embryonic and post-embryonic development of vertebrates by which the skeletal structures are formed. Several differences between mammals and teleosts regarding skeletal tissue types, their differentiation, remodeling and resorption of skeletal tissues have been reviewed in Boglione et al. (2013a). For instance, unlike mammalian species, skeletal growth in many teleost species continues throughout life (Witten and Huysseune, 2009); while fish larvae skeleton is less developed at birth (Haga et al., 2009). Despite these differences in the spatiotemporal formation, teleosts share all the same basic machinery (molecular mechanisms and cell types: chondrocytes, osteoblast, osteocytes and osteoclasts; with some exceptions regarding the presence of osteocytes) for bone development (Boglione et al., 2013a). Thus, they represent a suitable biological model for studying how different biotic and abiotic factors might affect skeletogenesis (Sire et al., 2009; Witten and Huysseune, 2009; Boglione et al., 2013b).

One of the major consequences of fish hatching at a much earlier developmental stage than other vertebrates is that nutrition, among other factors, plays a key role in controlling early fish development. Therefore, it can be experimentally modulated easily, in contrast to mammalian species where it can be hardly achieved due to regulation by the maternal metabolism. In this regard, a number of studies linked several nutrients with the skeletal phenotype when their level and/or form of supply in the diet were inappropriate or unbalanced (Boglione et al., 2013b). One of the most extensively studied nutrients is vitamin A (VA), a fat soluble vitamin that is not *de novo* synthesized by vertebrates (Ross et al., 2000) and thus, it has to be provided in a fine-tuned level and chemical form for proper vertebrate development. Several studies have described how fish larvae fed high levels of VA showed an abnormal skeletogenesis (Fernández and Gisbert, 2011; Boglione et al., 2013a). Nutritional VA requirements/effects seem to be cell/tissue, developmental stage and species-specific, but the particular signaling pathway by which retinoic acid (RA), the most active form of VA, induce abnormal skeletogenesis is still not fully understood.

At the nuclear level, RA is known as a ligand for the VA nuclear receptors [retinoic acid receptors (RARs) and retinoid X receptors (RXRs); Germain et al., 2006a,b] in order to control the transcription of genes involved in cell proliferation/differentiation and/or activity, and particularly that of chondrocytes, osteoblasts and/or osteoclasts. Some reports suggested that in fish RA is important for osteoblast (the bone forming cells) differentiation and function (Laue et al., 2008; Spoorendonk et al., 2008). Accordingly, Li et al. (2010) proposed two distinct roles of RA in the osteoblast lineage; an early role in blocking the recruitment of osteoblasts, and a later role in mature osteoblasts, promoting bone matrix synthesis. Additionally, in fish, bones formed through endochondral ossification were more prone to develop abnormalities than those ossifying through intramembranous ossification under a dietary VA imbalance, suggesting a physiological control by VA in both chondrocyte and osteoblast cells (Fernández and Gisbert, 2010). The disturbance of the RAR signaling pathway regulating osteoblast activity has been suggested to be the main cause of jaw deformities induced by VA imbalance in Japanese flounder (*Paralichthys olivaceus*) larvae when RXR and RAR selective agonists exposure was compared (Haga et al., 2002). More recently, we have shown that RARα seems to be the most specific VA pathway inducing abnormal skeletogenesis using *in vivo* and *in vitro* approaches in gilthead seabream (*Sparus aurata*) (Fernández et al., 2011, 2014). Nevertheless, skeletal deformities might be also indirectly induced through VA action on: (i) digestive system maturation, by perturbing nutrient uptake (Fernández et al., 2008); (ii) muscle development (Hamade et al., 2006), through the mechanostat theory (Fiaz et al., 2010); (iii) pituitary cells (Sternberg and Moav, 1999), by altering the synthesis and secretion of growth factors (Fernández et al., 2011); or (iv) thyroid follicles, by modifying homeostasis of thyroid hormones (THs; Fernández et al., 2009). The latter have been demonstrated to have a direct role on skeletal development and bone mineral density in mammals (reviewed in Gogakos et al., 2010), while in fish species it is known to control metamorphosis (Manchado et al., 2008a,b; Campinho et al., 2010; Gomes et al., 2015; Shao et al., 2016). The RA and THs signaling pathways crosstalk in flatfish metamorphosis (eye migration and adult pigmentation acquisition) has been recently hypothesized, being the basis for the generation of asymmetry in flatfish (Shao et al., 2016). However, little is known about the interaction between VA and TH signaling pathways on the skeletal development. Furthermore, such knowledge in mammalian species is still scarce. While VA impact on thyroid homeostasis has been reported *in vivo* (Silva et al., 2009; Mühlbauer et al., 2010) and *in vitro* (Fröhlich et al., 2004), only a recent study suggested that TH signaling might also impact VA signaling/homeostasis (Li et al., 2015).

Fish larvae undergo metamorphosis during their early ontogeny, a highly complex transition process that will lead larvae to become juveniles. This is an endocrine driven process in which THs play a central role (Pittman et al., 2013). In flatfish, this process involves a set of profound morphological, biochemical and physiological transformations, to transform them from a pelagic to a benthic mode of life (Geffen et al., 2007). Senegalese sole (*Solea senegalensis*) is a flatfish species considered a promising flatfish species for aquaculture production (Morais et al., 2016). Consequently, an increased understanding has been gained on the manner in which husbandry practices, environmental conditions, genetic background and/or nutrition requirements influence adult development. Previous results of our group showed how a dietary VA excess affected the number and size of thyroid follicles as well as the TH immunoreactivity (Fernández et al., 2009) and how exposure to RA signaling agonist and antagonist affected THs signaling at the transcriptional level (Boglino et al., 2017) during Senegalese sole metamorphosis. Nevertheless, how dietary VA imbalance during different phases of early fish development might affect juvenile skeletal phenotype and physiology through the interaction with THs signaling is still not known.

The objective of the present study was (i) to evaluate the effect of graded levels of dietary VA administered to Senegalese sole larvae during pre-, pro- and post-metamorphosis, with emphasis on its impact on thyroidal follicles and skeletal development; and (ii) to provide new insights into the underlying molecular mechanisms of abnormal skeletogenesis under dietary VA imbalance.

## Materials and methods

### Ethics statement

All experiments complied with the ARRIVE guidelines (Kilkenny et al., 2010) and were performed according to 2010/63/EU of the European Parliament and Council, and to guideline 86/609/EU of the European Union Council. Animal experimental procedures were conducted in compliance with the experimental research protocol (reference number 4978-T9900002) approved by the Committee of Ethic and Animal Experimentation of the IRTA and the Departament de Medi Ambient i Habitatge (DMAH, Generalitat de Catalunya, Spain). For sampling purposes, soles were sacrificed with an overdose of anesthetic (Tricaine methanesulfonate, MS-222, Sigma-Aldrich).

### Larval rearing and experimental design

Newly hatched larvae were distributed (initial density: 80 larvae L^−1^) in 21 cylindrical tanks (100 L) connected to a recirculation unit (IRTAmar™). Water conditions were as follows: 18.0 ± 1.0°C, 35 ppt salinity, pH between 7.8 and 8.2, and daily exchange of water (20%) in the recirculation system with gentle aeration and oxygenation (>4 mg L^−1^). Photoperiod was 12 L:12 D and light intensity was 500 lx at water surface.

General feeding protocol for Senegalese sole used in the present study was as follows: pre-metamorphic larvae were fed from 3 days post hatch (dph) to 10 dph with rotifers (*Brachionus plicatilis*) enriched with Easy Selco™ (ES, INVE, Belgium). Rotifer density in larval rearing tanks was 10 rotifers mL^−1^ from 3 to 6 dph and gradually reduced to 5 rotifers mL^−1^ at 10 dph. Rotifer density was adjusted twice a day in order to assure the optimal prey density. Enriched *Artemia* metanauplii (EG, INVE, Belgium) were offered to sole from 6 to 40 dph at increasing densities from 0.5 to 12 metanauplii mL^−1^. *Artemia* metanauplii density was adjusted four times per day (at 9, 12, 15, and 18 h) to assure the optimal prey density and nutritional VA value, as described in Cañavate et al. (2006). No changes in the levels of VA content in enriched *Artemia* metanauplii were observed during the first 4 h post-enrichment in larval rearing tanks (Fernández, unpublished data). From 20 dph onwards, when individuals showed completion of eye migration and begin to show a benthonic behavior, the volume of rearing tanks was reduced to 40 L and enriched *Artemia* was delivered frozen. From 41 dph to the end of the experiment (55 dph), post-metamorphic larvae were weaned onto dry feed (Gemma Micro 150–300^©^ Skretting, Spain).

**Figure 1:**
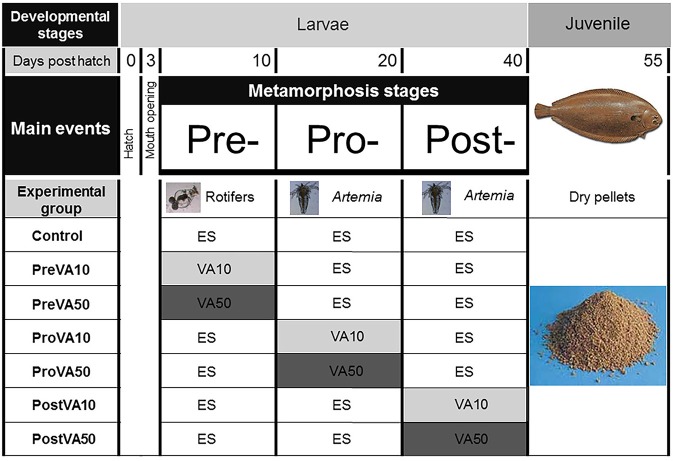
Experimental design of the present study showing from top to bottom: (i) *Developmental stages* considered (*larvae* and *juvenile*); (ii) *Days post hatch* (*0, 3, 10, 20, 41*, and *55*); (iii) *Main events* during larval development (*hatch, mouth opening, pre-metamorphosis, pro-metamorphosis, post-metamorphosis*); (iv) *Experimental groups* listed on the left column, graphically depicted in central columns: *Control*, larvae fed rotifers and *Artemia* metanauplii enriched with Easy Selco (ES); *PreVA10*, larvae fed rotifers enriched with ES supplemented with a dietary content 10 fold above normal (VA10) and afterwards *Artemia* metanauplii enriched with ES; *PreVA50*, larvae fed rotifers enriched with ES supplemented with a dietary content 50 fold above normal (VA50) and afterwards *Artemia* metanauplii enriched with ES; *ProVA10*, larvae fed rotifers enriched with ES, *Artemia* metanauplii enriched with ES supplemented with a dietary content in 10 fold above normal (VA10) during pro-metamorphosis (from 10 to 20 dph) and afterwards *Artemia* metanauplii enriched with ES; *ProVA50*, larvae fed rotifers enriched with ES, *Artemia* metanauplii enriched with ES supplemented with a dietary content in 50 fold above normal (VA50) during pro-metamorphosis (from 10 to 20 dph) and afterwards *Artemia* metanauplii enriched with ES; *PostVA10*, larvae fed rotifers enriched with ES, with *Artemia* metanauplii enriched with ES (from 10 to 20 dph) and afterwards *Artemia* metanauplii enriched with ES supplemented with a dietary content in 10 fold above normal (VA10) during post-metamorphosis (from 21 to 41 dph); *PostVA50*, larvae fed rotifers enriched with ES, with *Artemia* metanauplii enriched with ES (from 10 to 20 dph) and afterwards *Artemia* metanauplii enriched with ES supplemented with a dietary content in 50 fold above normal (VA50) during post-metamorphosis (from 21 to 40 dph); and weaning process for all experimental groups on the right column from 41 to 55 dph.

To evaluate the effect of VA on Senegalese sole development, three different dietary VA levels (non-supplemented, 10 and 50 times supplemented; denoted as ES, VA10, VA50) were supplied and performed in triplicate at three clearly defined developmental stages: pre-, pro- and post-metamorphosis, whereas the rest of the time, fish were fed with the non-enriched VA live prey (ES), as shown in Figure 1. Imbalance on dietary VA content was induced by the addition of retinyl palmitate (1,600,000 IU g^−1^, Sigma-Aldrich, Spain) to the Easy Selco™ emulsion; enriching emulsions supplemented with VA (VA10 and VA50) or not (ES) were used to enrich both live preys (carriers), rotifers during pre-metamorphosis and *Artemia* metanauplii during pro- and post-metamorphosis. Both live preys were enriched as previously described in Fernández et al. (2008).

### Biochemical analyses

The retinoid content of enrichment emulsions, enriched live prey, and sole were analyzed by HPLC, using a modified version of the method by Takeuchi et al. (1998). After sampling, live prey and larvae were washed with distilled water to remove salt and bacteria, and the samples were frozen at −80°C until posterior analysis. Experimental enriching emulsions (ES, VA10, and VA50) and enriched live prey were analyzed in triplicate. For retinoid content in sole, samples (1 g wet weight) were taken at 10, 21, 41, and 55 dph from each tank/replicate (three biological replicates). Lipids were extracted with chloroform:methanol (C:M, 2:1) according to Folch's method (Folch et al., 1957) and stored in C:M:BHT (2:1:0.01%) at 20 mg L^−1^ and −20°C until analysis. Lipid extracts were then evaporated and dissolved in methanol:acetone (1:1, v/v) prior to retinoid HPLC analysis. The HPLC system (Thermo Separation Products, San Jose, CA, USA) was equipped with a Lichrospher C-18 reversed-phase column (250 mm length and 4 mm diameter; Merck, Darmstadt, Germany) and a UV–visible detector set at a wave length of 325 nm, and retinoid determination was performed as in Fernández et al. (2009). No gradient was used. The mobile phase was a mixture (85:15, v/v) of methanol (98%) with 0.5% ammonium acetate and chloroform. The flow rate was 1.5 ml min^−1^, and the elution time was 18 min. The specificity of the method for the different retinoid compounds is guaranteed by the retention times of the peaks in the standard injections and the lack of interfering peaks in the blank runs. The standards used and the relative retention times were: retinoic acid (Sigma-Aldrich R-2625; 1.86 min), retinol (Sigma-Aldrich R-7632; 2.29 min), all-trans retinal (Sigma-Aldrich R-2500; 2.64 min), retinyl palmitate (Sigma-Aldrich R-3375; 14.58 min), and the internal standard retinyl acetate (Sigma-Aldrich R-4632; 3.14 min). In analyzed samples, retention times were 1.91, 2.27, 2.69, 14.66, and 3.13 min, respectively (Supplementary Figure 1). The four point linear regressions of the peak area and the concentration ratios of the internal standard and each retinoid analyzed had *r*^2^ higher than 0.9886, and were considered linear in the range of the tested samples. The repeatability was assessed through the injection of five different standard solutions (Sigma-Aldrich) with a mixture of the retinoids analyzed for each of the four levels used in the calibration curves. The coefficient of variation was in all cases below 5%. These standard analyses also allowed checking the % recovery of the assayed retinoids, which was found between 92 and 101%. No peak was considered below a signal/noise ratio of 10.

### Monitoring of growth, survival, and metamorphosis

At 10, 21, 41, and 55 dph, 15 individuals were randomly sampled from each tank, rinsed with distilled water, and used for standard length (SL) and dry weight (DW) determination. Larval width was only evaluated in fully metamorphosed and bottom-settled individuals (55 dph). Larval standard length (SL) and width was measured with a digital camera connected to a binocular microscope (Nikon SMZ 800) and an image analysis system (AnalySIS, Soft Imaging Systems, GmbH). After sole were used for morphometric purposes, they were dried at 60°C until their weight was constant. DW of samples was assessed in an analytic microbalance (Sartorius BP211D). Final survival rate was calculated as the percentage of final surviving fish with respect to the initial number at the beginning of the trial minus those individuals removed for sampling.

The eye migration index is generally used in Senegalese sole as a measure of metamorphosis progress. Eye migration was evaluated at 10, 21, 41, and 55 dph (30 specimens per replicate) as in Fernández-Díaz et al. (2001), and data were expressed as the relative amount (%) of specimens at each developmental stage at the same sampling time (age).

### Mineralization degree and skeletal phenotype analyses

To evaluate the mineralization degree of the skeleton, and to identify and quantify the incidence of skeletal deformities, 30 specimens per tank were sampled at 10, 21, and 55 dph and fixed in formaldehyde solution (10%). Animals were stained with Alizarin red for bone and Alcian blue for cartilage in whole mount preparations using a modification of the method described by Klymkowsky and Hanken (1991). Skeletal structures were identified and named according to Okada et al. (2001), Wagemans and Vandewalle (2001) and Gavaia et al. (2002). At 10, 21, and 55 dph, the mineralization degree (non-, slightly-, or fully-mineralized), meristic characters and skeletal abnormalities in the cranium, vertebral column and caudal fin complex were evaluated (for a detailed view of skeletal structures see Supplementary Figure 2).

### Thyroid gland development

The development of the thyroid gland (number and size of thyroidal follicles) as well as detection and semiquantification of THs, thyroxin (T4) and triiodothyronine (T3) were evaluated on histological sections of fish aged 10, 21, and 55 dph (*n* = 3 specimens per rearing tank; *n* = 9 per dietary treatment) accordingly to Ortiz-Delgado et al. (2006) and using monoclonal antibodies (Cat. 10-T35A M94210-515 and Cat. 10-T30B M94207-3010 from Fitzgerald (USA), respectively). Three different thyroid follicle regions/contents were considered for the evaluation of THs immune-reactivity (Supplementary Figure 3).

### Gene expression analyses

Total RNA was extracted from pools of whole body specimens (50 to 3 individuals per sample at 6, 10, 21, and 41 dph depending on fish size) using TRIzol reagent (Invitrogen®, San Diego, CA, USA) following manufacturer's protocol. RNA was quantified using a Gene-Quant spectrophotometer (Amersham Biosciences) and purity established by the absorbance ratio 260/280 nm. The integrity of the RNA was examined by gel electrophoresis. Total RNA (1 μg) was retrotranscribed using the QuantiTect Reverse Transcription Kit (Qiagen®); electrophoresis using a 1.2% agarose gel was run to assess the specificity of RT-PCR product. Real-time qPCR was performed using an ABI PRISM 7300 (Applied Biosystems). For each gene, a species-specific Taqman assay was designed (Applied Biosystems) using the sequences acquired from the GenBank database (Supplementary Table 1). The efficiency of the Taqman assay for each gene was previously evaluated to assure that it was close to 100%. All reactions were performed in 96 well plates in triplicate in 20 μl reaction volumes containing: 10 μl of 2× TaqMan universal PCR master mix (Applied Biosystems); 1 μl of the 20× Taqman primer/probe solution corresponding to the analyzed gene; 8 μl of molecular biology grade water; and 1 μl of cDNA diluted 1:10, with the exception of *bone Gla protein* (*bgp*), which was evaluated with a 1:5 dilution. Standard amplification parameters were as follows: 95°C for 10 min, followed by 45 amplification cycles, each of which comprised 95°C for 15 s and 60°C for 1 min. Real time qPCR was performed for each gene following MIQE guidelines such as including a calibrator sample within each plate (Bustin et al., 2009). The relative gene expression ratio for each gene was according to Pfaffl (2001). Relative gene expression was normalized using *ubiquitin* (*ubq*), a previously reported reference gene for accurate normalization in qPCR studies with Senegalese sole (Infante et al., 2008; Richard et al., 2014; Fernández et al., 2015).

### Statistical analyses

Results are given as mean and standard deviation. Data expressed as percentage (survival, incidence of skeletal deformities, eye migration success, and mineralization degree) were previously arcsin(x^1/2^)-transformed. All data were checked for normality (Kolmogorov–Smirnov test) and homoscedasticity of variance (Bartlett's test) and then compared by means of One Way ANOVA for multiple comparisons. When significant differences were detected, Tukey multiple-comparison test was used to detect differences among experimental groups. The level of significant difference was set at *P* < 0.05. All the statistical analyses were conducted using GraphPad Prism 5.0 (GraphPad Software, Inc.).

## Results

### Biochemical analyses

#### Lipids and retinoid levels in experimental emulsions and enriched live prey

Total lipid levels and retinoid content in the experimental emulsions (ES, VA10, and VA50), enriched rotifers and *Artemia* metanauplii are presented in Table 1. Lipid content was not significantly affected by retinyl palmitate addition neither in the experimental emulsions nor in the enriched live prey (ANOVA, *P* > 0.05).

**Table 1:** Total lipid and retinoids (mean values ± standard deviation) content in experimental enriching emulsions, enriched rotifers and *Artemia* metanauplii.

	**Experimental group**	**Lipid content (mg g^−1^ DW)**	**RA content (IU Kg^−1^ DW)**	**Retinal content (IU Kg^−1^ DW)**	**Retinol content (IU Kg^−1^ DW)**	**Retinyl palmitate content (IU Tn^−1^ DW)**	**Total VA content (IU Tn^−1^ DW)**
Enriching emulsion	ES	689.80 ± 10.42	62.95 ± 16.72^c^	0.69 ± 0.07^b^	19.12 ± 1.49^b^	*1, 291.85* ± 86.32^c^	*1, 291.93* ± 86.32^c^
	VA10	693.0 ± 13.17	225.08 ± 4.61^b^	0.89 ± 0.1^a^	10.51 ± 3.1^b^	*19, 897.94* ± *3, 173.22*^b^	*19, 898.18* ± 3, 173.22^b^
	VA50	711.77 ± 57.86	574.5 ± 112.75^a^	0.0 ± 0.0^c^	75.95 ± 9.65^a^	*57, 220.82* ± *8, 326.61*^a^	*57, 221.47* ± *8, 326.63*^a^
Enriched rotifers	ES	75.25 ± 1.52	*n.d*.	*n.d*.	16.14 ± 5.53^c^	31.39 ± 3.5^c^	31.41 ± 3.5^c^
	VA10	90.02 ± 10.23	55.5 ± 10.68^b^	*n.d*.	365.75 ± 20.9^b^	264.15 ± 70.2^b^	264.52 ± 70.18^b^
	VA50	84.62 ± 9.73	216.75 ± 23.3^a^	*n.d*.	*1, 635.33* ± 145.85^a^	912.67 ± 55.27^a^	914.31 ± 55.42^a^
Enriched *Artemia* metanauplii	ES	135.80 ± 17.87	*n.d*.	*n.d*.	12.48 ± 1.55^c^	4.23 ± 0.44^c^	4.25 ± 0.45^c^
	VA10	131.54 ± 21.2	96.25 ± 10.25^b^	*n.d*.	59.62 ± 2.61^b^	12.7 ± 0.04^b^	12.85 ± 0.03^b^
	VA50	134.1 ± 18.89	*3, 245.56* ± 301.27^a^	*n.d*.	328.36 ± 30.94^a^	415.64 ± 58.55^a^	419.21 ± 58.73^a^

*Total lipid content determined in enriching emulsions (N = 3), enriched rotifers (N = 3) and enriched Artemia metanauplii (N = 3). Retinoid and total retinoid content determined in enriching emulsions (N = 3), enriched rotifers (N = 3), and enriched Artemia metanauplii (N = 3). Total VA, total retinoid content; ES, easy selco; VA10, ES supplemented its retinoid dietary content in 10 times; VA50, ES supplemented its retinoid dietary content in 50 times; n.d., non-detected. Different superscript letters within the column of each parameter evaluated and enriching emulsion, enriched rotifers or enriched Artemia metanauplii denotes statistically significant differences among experimental groups (ANOVA, P < 0.05)*.

In general, retinoid content was significantly increased in experimental emulsion by retinyl palmitate addition, and particularly regarding the retinoic acid (RA), retinyl palmitate and total VA (sum of the content of all the retinoids: RA, retinal, retinol and retinyl palmitate) content (ANOVA, *P* < 0.05). Retinal was found in experimental emulsions in a very low level while not detected in enriched live preys. Retinol content was significantly increased in experimental emulsions and enriched live preys, with the unique exception of ES and VA10 emulsions.

#### Lipids and retinoid levels in fish

Total lipid content in larvae was found homogeneous among the different sampling times (10, 21, 41, and 55 dph), even when soles were fed increasing levels of VA (Table 2), with the exception of day 21 in which specimens from the PreVA50 group showed a higher total lipid content (13.77 ± 1.65 mg g^−1^ DW) than that of PreVA10 group (8.44 ± 0.18 mg g^−1^ DW; ANOVA, *P* < 0.05).

**Table 2:** Total lipid and retinoid (mean ± SD) content in Senegalese sole individuals at 10, 21, 41, and 55 dph fed experimental diets.

**Experimental group**	**dph**	**Lipid content**	**Retinol content**	**Retinyl palmitate content**	**Total VA content**
Control	10	10.19 ± 0.24	7.39 ± 0.45^b^	*9, 882.67* ± *1, 656.59*^c^	*9, 890.05* ± 1, 657.05^c^
PreVA10		9.6 ± 1.41	13.46 ± 1.06^b^	*24, 422.33* ± *1, 686.99*^b^	*24, 435.8* ± *1, 687.64*^b^
PreVA50		10.89 ± 3.86	50.45 ± 11.54^a^	*121, 165.0* ± *11, 749.29*^a^	*121, 215.45* ± *11, 760.83*^a^
Control	21	12.15 ± 0.15^a, b^	4.76 ± 0.72^c, d^	*11, 373.0* ± 688.72^b, c^	*11, 377.76* ± 689.44^b, c^
PreVA10		8.44 ± 0.18^b^	3.39 ± 0.01^d^	*8, 452.0* ± 591.14^c^	*8, 455.39* ± 591.13^c^
PreVA50		13.77 ± 1.65^a^	6.58 ± 0.99^b, c^	*20, 752.0* ± *5, 583.31*^b^	*20, 758.58* ± *5, 584.31*^b^
ProVA10		11.59 ± 1.38^a, b^	5.37 ± 0.56^b, c, d^	*14, 898.0* ± *1, 845.55*^b, c^	*14, 903.37* ± *1, 846.11*^b, c^
ProVA50		10.99 ± 2.02^a, b^	10.14 ± 0.95^a^	*36, 373.5* ± *1, 950.91*^a^	*36, 383.64* ± *1, 949.95*^a^
Control	41	12.04 ± 1.65	1.59 ± 0.66^b^	*8, 975.5* ± 152.03^b^	*8, 977.09* ± 152.69^b^
PreVA10		11.0 ± 0.37	3.47 ± 0.61^b^	*13, 354.33* ± 852.88^b^	*13, 357.8* ± 852.71^b^
PreVA50		11.55 ± 2.05	3.32 ± 0.67^b^	*13, 512.67* ± *4, 357.34*^b^	*13, 515.99* ± *4, 357.92*^b^
ProVA10		8.86 ± 1.01	3.28 ± 0.43^b^	*12, 741.67* ± *2, 491.01*^b^	*12, 744.94* ± *2, 491.33*^b^
ProVA50		9.83 ± 0.64	3.36 ± 0.65^b^	*15, 784.67* ± *1, 984.83*^b^	*15, 788.03* ± *1, 985.46*^b^
PostVA10		10.41 ± 1.11	3.42 ± 0.49^b^	*16, 277.0* ± *1, 873.79*^b^	*16, 280.42* ± *1, 874.08*^a^
PostVA50		9.49 ± 1.52	7.21 ± 1.97^a^	*41, 934.0* ± *7, 873.65*^a^	*41, 941.21* ± *7, 875.58*^a^
Control	55	12.25 ± 1.22	3.32 ± 1.43	*15, 695.5* ± *727.61*^b, c^	*15, 698.82* ± 729.04^b, c^
PreVA10		11.65 ± 0.17	3.3 ± 0.25	*10, 790.0* ± *3, 785.22*^c^	*12, 908.45* ± *1, 347.7*^c^
PreVA50		12.68 ± 0.92	3.64 ± 0.38	*15, 388.0* ± 314.89^b, c^	*15, 391.64* ± 315.27^b, c^
ProVA10		13.33 ± 1.11	4.09 ± 0.98	*19, 322.33* ± *4, 131.96*^a, b^	*19, 326.43* ± *4, 132.94*^a, b^
ProVA50		13.50 ± 0.32	3.42 ± 0.42	*16, 658.33* ± *1, 553.96*^b, c^	*16, 661.75* ± *1, 553.83*^b, c^
PostVA10		11.57 ± 0.61	4.14 ± 0.73	*19, 596.67* ± *3, 209.24*^a, b^	*19, 600.81* ± *3, 209.86*^a, b^
PostVA50		11.93 ± 0.52	3.85 ± 0.75	*25, 876.67* ± *1, 890.37*^a^	*25, 880.52* ± *1, 891.05*^a^

*Total lipid (mg g^-1^ DW), retinyl, retinyl palmitate and total VA (IU Kg^-1^ DW) content determined in Senegalese sole larvae fed live preys enriched with different experimental emulsions (ES, VA10 and VA50) at different developmental stages (pre-, pro- and post-metamorphosis; N = 3). Total VA, total retinoid content; Control, larvae fed rotifers and Artemia metanauplii enriched with Easy Selco (ES); PreVA10, larvae fed rotifers enriched with ES supplemented its dietary content in 10 times (VA10) and afterwards Artemia metanauplii enriched with ES; PreVA50, larvae fed rotifers enriched with ES supplemented its dietary content in 50 times (VA50) and afterwards Artemia metanauplii enriched with ES; ProVA10, larvae fed rotifers enriched with ES, then Artemia metanauplii enriched with ES supplemented its dietary content in 10 times (VA10) during pro-metamorphosis (from 10 to 20 dph) and afterwards Artemia metanauplii enriched with ES; ProVA50, larvae fed rotifers enriched with ES, with Artemia metanauplii enriched with ES supplemented its dietary content in 50 times (VA50) during pro-metamorphosis (from 10 to 20 dph) and afterwards Artemia metanauplii enriched with ES; PostVA10, larvae fed rotifers enriched with ES, then Artemia metanauplii enriched with ES (from 10 to 20 dph) and afterwards Artemia metanauplii enriched with ES supplemented its dietary content in 10 times (VA10) during post-metamorphosis (from 21 to 37 dph); PostVA50, larvae fed rotifers enriched with ES, thenwith Artemia metanauplii enriched with ES (from 10 to 20 dph) and afterwards with Artemia metanauplii enriched with ES supplemented its dietary content in 50 times (VA50) during post-metamorphosis (from 21 to 37 dph). Different superscript letters within the column of each parameter evaluated denote statistically significant differences among experimental groups (ANOVA, P < 0.05)*.

Regarding retinoid content, neither RA nor retinal were detected in any experimental group regardless of the sampling time. At the end of each metamorphic stage, larvae fed the highest VA level (PreVA50, ProVA50 and PostVA50) also showed the highest retinol content (50.45 ± 11.54, 10.14 ± 0.95, and 7.21 ± 1.97 IU Kg^−1^ DW at 10, 21, and 41 dph, respectively; ANOVA, *P* < 0.05). However, retinol content in post-metamorphic larvae aged 55 dph did not show significant differences, ranging from 3.3 ± 0.25 to 4.14 ± 0.73 IU Kg^−1^ DW (ANOVA, *P* < 0.05). Regarding retinyl palmitate body content, the same pattern was observed. Fish fed with the highest VA content during each developmental stage also showed the highest retinyl palmitate content at 10, 21, and 41 dph (ANOVA, *P* < 0.05). However, unlike retinol content, fish still exhibited significant differences in retinyl palmitate content at 55 dph (ANOVA, *P* < 0.05).

### Larval growth, survival rate, and metamorphosis

**Figure 2:**
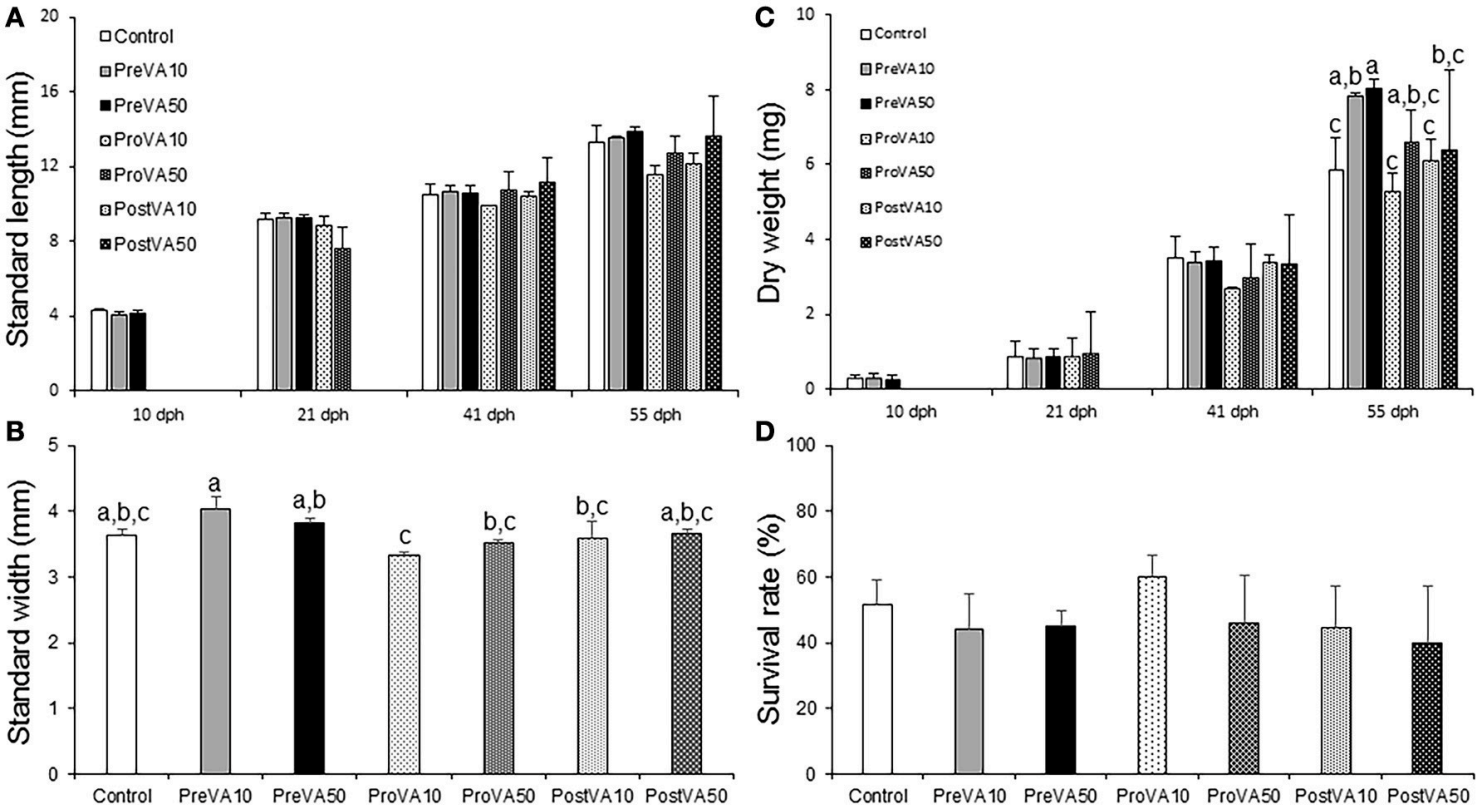
Larval performance (growth and survival; mean ± standard deviation) in Senegalese sole fed the different experimental diets. Standard length at 10, 21, 41, and 55 dph **(A)**, standard width at 55 dph **(B)**, dry weight at 10, 21, 41, and 55 dph **(C)** and survival rate at 55 dph. For a detailed description about the different experimental groups, please see the legend of Figure 1. Different letters at the top of each bar denote statistically significant differences among experimental groups (ANOVA, *P* < 0.05; *N* = 3).

The dietary VA level did not significantly affect Senegalese sole SL at any developmental stage (Figure 2A; ANOVA, *P* > 0.05). However, at the end of the trial (55 dph), juveniles fed increasing levels of VA showed significant differences in body width (Figure 2B; ANOVA, *P* < 0.05). Fish from the PreVA10 and ProVA10 groups showed the largest (0.4 ± 0.02 mm) and lowest (0.33 ± 0.01 mm) width values, respectively; whereas the rest of the groups showed intermediate values ranging from 0.35 ± 0.01 to 0.38 ± 0.01 mm.

Growth as determined by DW was also affected by dietary VA levels and metamorphic stages, but only at the end of the trial (55 dph; Figure 2C). Sole from PreVA10 and PreVA50 groups showed the highest values (7.83 ± 0.45 and 8.04 ± 0.12 mg, respectively). Fish from the Control, ProVA10 and PostVA10 groups displayed the lowest values (between 5.26 ± 0.94 and 6.4 ± 0.23 mg; ANOVA, *P* < 0.05). Finally, survival rates (between 40 and 60%, Figure 2D) and percentage of sole with specific eye migration index (results not shown) were similar among experimental groups (ANOVA, *P* > 0.05).

### Degree of skeletal mineralization and incidence of skeletal deformities

**Figure 3:**
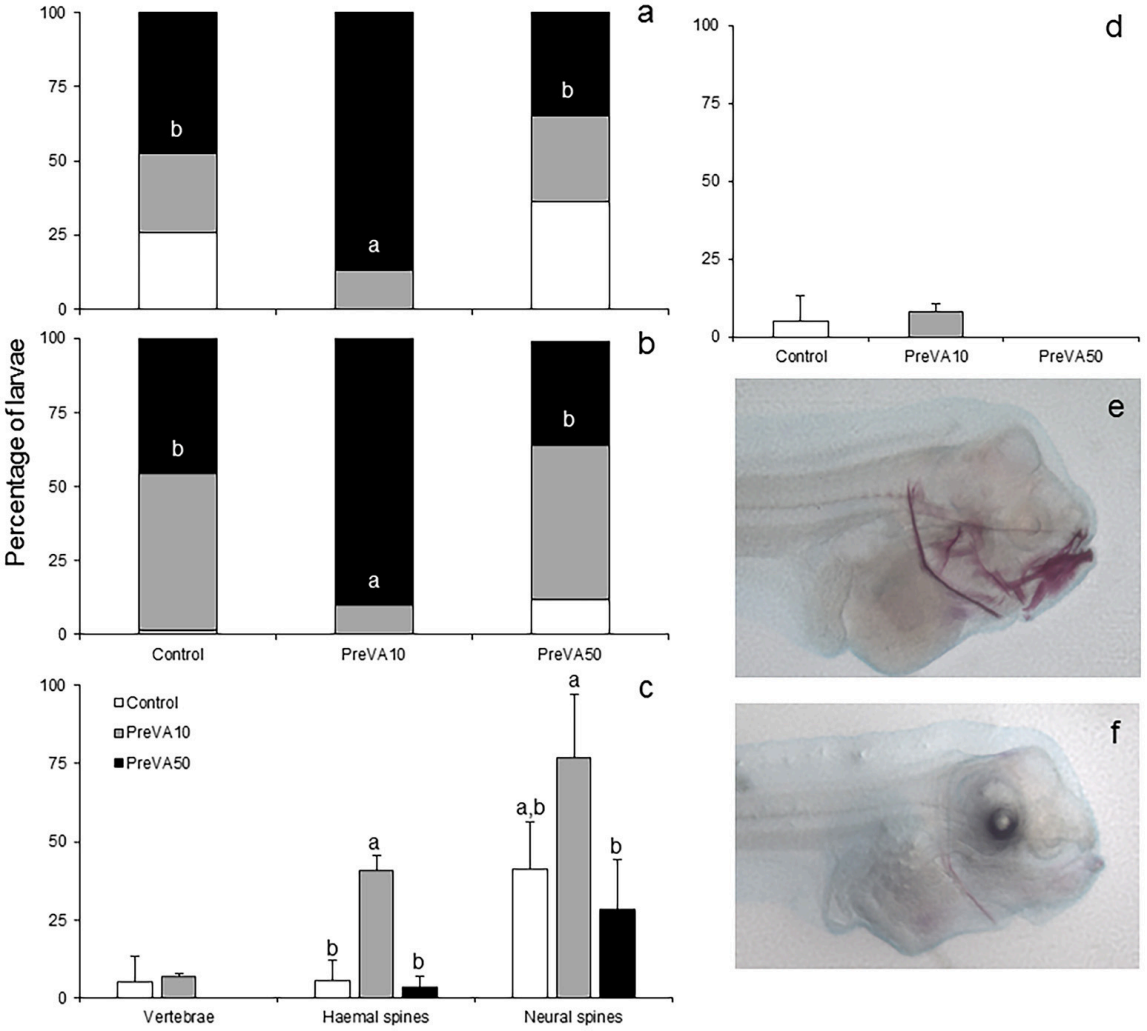
Mineralization degree of different skeletal structures in 10 dph Senegalese sole larvae fed increased dietary vitamin A levels at different developmental stages. Percentage of larvae showing different degrees of mineralization showing fully mineralized (black bar fraction), slightly mineralized (gray bar fraction) and not mineralized (white bar fraction) structures of upper jaw **(a)** and lower jaw **(b)**. Percentage of larvae showing an onset of mineralization in axial structures (vertebrae, haemal and neural spines) **(c)**, and hypurals **(d)**. Detailed view of the degree of mineralization of several cranial structures from PreVA10 **(e)** and Control **(f)** larvae at 10 dph. Note the extended alizarin red staining in several structures (upper and lower jaws, cleithrum, ethmoid and neural spines) in the PreVA10 larvae, whereas a slight mineralization of the cleithrum in the Control larvae. For a detailed description about the different experimental groups, please see the legend of Figure 1. Different letters within black bar fraction in graphs **(a,b)** or at the top of each bar on the rest of the graphs denote statistically significant differences among experimental groups (ANOVA, *P* < 0.05; *N* = 3).

At 10 dph, different VA dietary regimes provided at pre-metamorphosis already induced a significantly different degree of mineralization in cranial, axial and caudal structures (Figure 3). PreVA10 larvae showed a higher percentage of fully mineralized upper and lower jaws than in the rest of experimental groups (Figures 3a,b,e,f; ANOVA, *P* < 0.05). Similar results were also observed in other cranial structures such as the cleithrum, branchial arches, and ethmoid (results not shown). Similarly, such VA regimes also significantly affected the mineralization degree of some axial structures (Figure 3c; ANOVA, *P* < 0.05) such as the haemal and neural spines, but not the percentage of pre-metamorphic larvae with ossified vertebrae nor the hypurals at the caudal fin complex (Figure 3d; ANOVA, *P* > 0.05).

**Figure 4:**
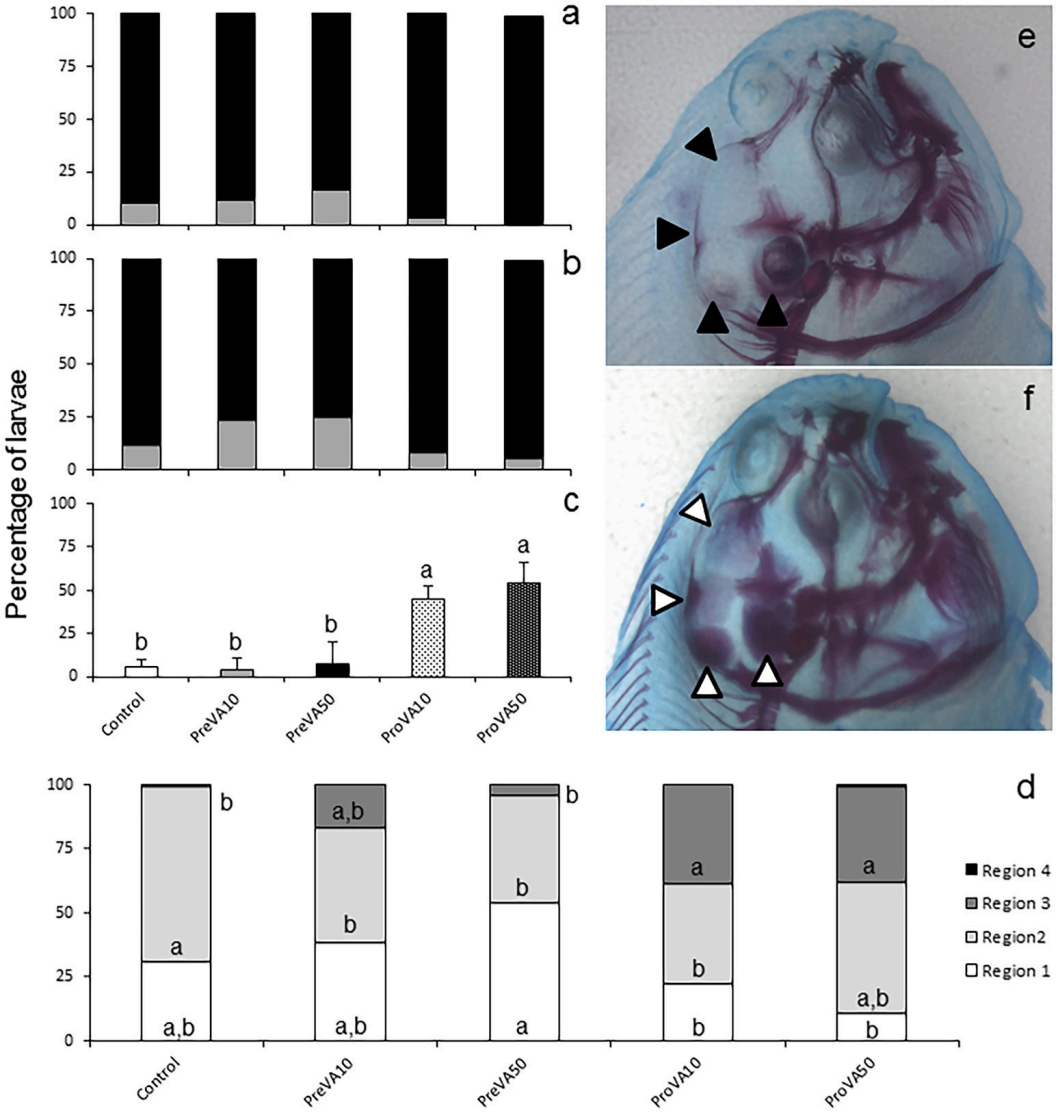
Mineralization degree of different skeletal structures in 21 dph Senegalese sole larvae fed with increased dietary vitamin A levels at different developmental stages. Percentage of larvae showing different degrees of mineralization (showing fully mineralized (black bar fraction), slightly mineralized (gray bar fraction) and not mineralized (white bar fraction) structures) of upper jaw **(a)** and lower jaw **(b)**. Percentage of larvae showing an onset of mineralization in closely related axial structures (hyoid, interhyal, pre-opercular and quadrate) **(c)**, and in defined cranial regions (R1, R2, R3, and R4; please see further information in Supplementary Figure 2) **(d)**. A detailed view of the degree of mineralization of several cranial structures from Control **(e)** and ProVA50 **(f)** larvae at 21 dph is presented. Note the extended alizarin red staining in several cranial regions when comparing Control (black arrow heads) with ProVA50 (white arrow heads) larvae. For a detailed description about the different experimental groups, please see the legend of Figure 1. Different letters within white, light gray, strong gray and black bar fraction in graph **(d)** or at the top of each bar in graph **(c)** denotes statistically significant differences among experimental groups (ANOVA, *P* < 0.05; *N* = 3).

At 21 dph, VA dietary regimes provided at pre- and pro-metamorphosis significantly affected the degree of mineralization, particularly in cranial, axial and caudal structures (Figure 4 and Supplementary Figure 4). No differences in the degree of mineralization of upper and lower jaws were found in specimens from the different experimental groups (Figures 4a,b), neither regarding the percentage of larvae with mineralized vertebrae (Supplementary Figure 4a), nor the haemal and neural spines (ANOVA, *P* > 0.05; results not shown). However, a distinct mineralization degree was found in cranial structures such as the complex formed by the hyoid, interhyal, pre-opercular, and quadrate (Figure 4c) and different regions of the neurocranium (R_1_, R_2_, R_3_, and R_4_; Supplementary Figure 1). A higher percentage of soles from ProVA10 and ProVA50 groups with an earlier onset of mineralization at the hyoid, interhyal, pre-opercular, and quadrate was found compared to that of Control, PreVA10 and PreVA50 specimens (ANOVA, *P* < 0.05). Similarly, ProVA10 and ProVA50 groups showed the highest advanced mineralization degree in the cranium (37.06 and 38.49% in the R_3_ area, respectively; Figures 4e,f). In addition, although not significantly different, ProVA10 and ProVA50 groups tended to show a higher percentage of specimens with mineralized modified neural spines (Mns), epural and hypurals (1-5) (Supplementary Figures 4b–d; ANOVA, *P* > 0.05). A higher mean number of formed caudal rays (Supplementary Figure 4e; ANOVA, *P* < 0.05) was observed in fish from PreVA10, ProVA10, and ProVA50 groups compared to those of the PreVA50 group, whereas specimens from the Control group had intermediate values. Furthermore, some skeletal deformities in the caudal fin region were already clearly identified at 21 dph (Supplementary Figures 4f,g).

**Figure 5:**
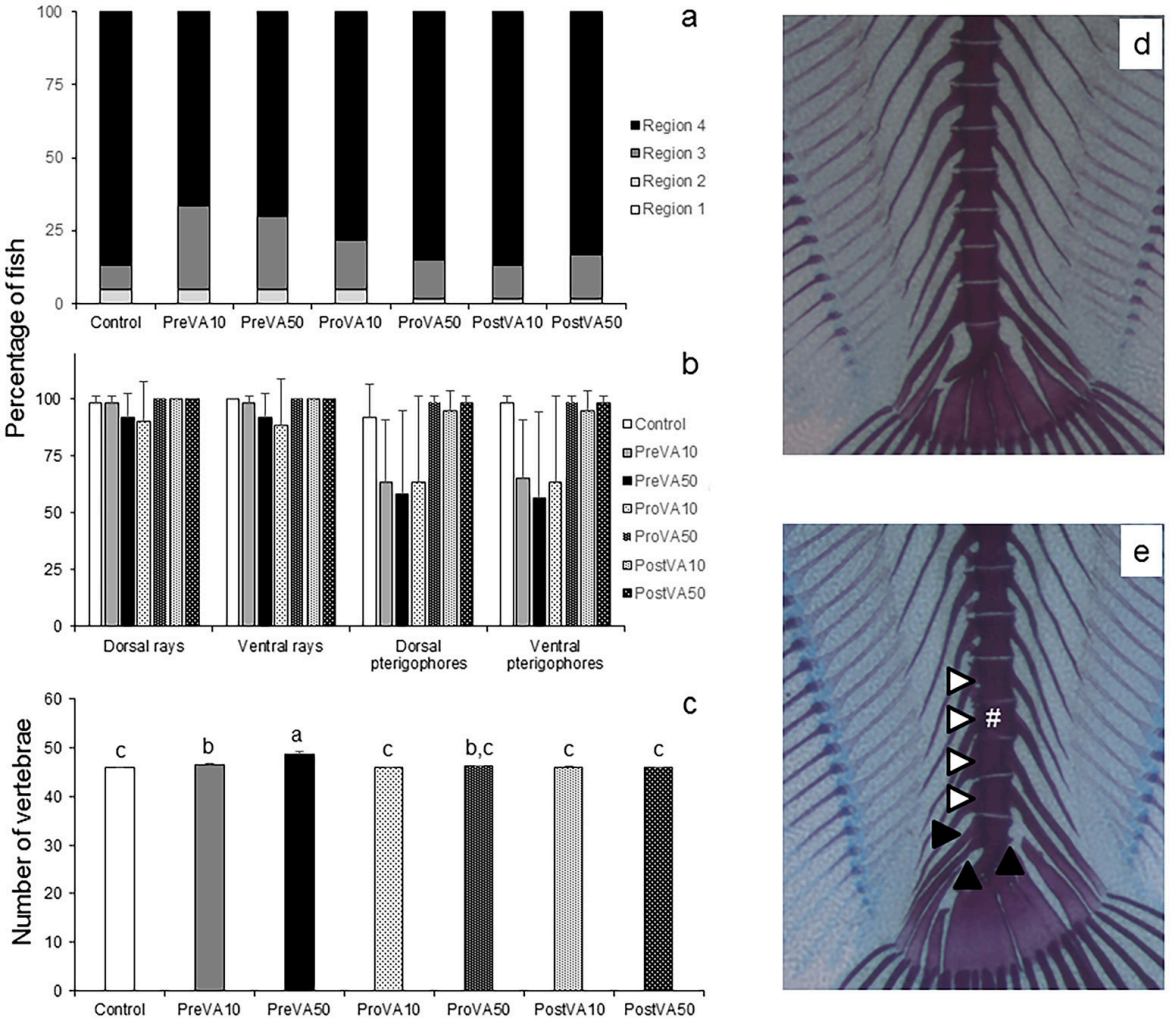
Mineralization degree of different skeletal structures and mean number of vertebrae in 55 dph Senegalese sole larvae fed increased dietary vitamin A levels at different developmental stages. Percentage of larvae showing mineralization in cranial regions (R1, R2, R3, and R4; please see further information in Supplementary Figure 2) **(a)**, dorsal and ventral rays and pterigophores **(b)** and mean number of vertebrae **(c)**. A detailed view of the caudal fin complex from Control **(d)** and PreVA50 **(e)** fish at 55 dph is presented. Note the additional presence of a preural vertebrae (white #) and the related vertebral compression in preural vertebrae (white arrowheads), and the presence of neural and haemal spines in the urostyle (black arrowheads) in PreVA50 fish compared with the Control fish. For a detailed description about the different experimental groups, please see the legend of Figure 1. Different letters at the top of each bar in graph **(c)** denote statistically significant differences among experimental groups (ANOVA, *P* < 0.05; *N* = 3).

At the end of the trial (55 dph), Senegalese sole early juveniles fed different dietary VA levels at different metamorphic stages have mineralized all the skeletal elements with some particular exceptions for R_2_, R_3_, and R_4_ regions. Nevertheless, no significant differences were found between experimental groups when the percentage of specimens with mineralized R_2_, R_3_, or R_4_ region were considered (Figure 5a; ANOVA, *P* > 0.05). Similarly, the percentage of fish with mineralized neural rays, ventral rays or pterigophores was also not significantly different among experimental groups (Figure 5b; ANOVA, *P* > 0.05). Finally, fish from PreVA50 had the highest mean number of vertebrae (48.68 ± 0.4), followed by fish from PreVA10 group (46.57 ± 0.12). ProVA50 fish showed intermediate values (46.08 ± 0.02) while those from Control, ProVA10, PostVA10 and PostVA50 groups showed the lowest number (ranging from 45.85 ± 0.2 to 46.0 ± 0.08; Figure 5c; ANOVA, *P* < 0.05). Other minor abnormalities such as the presence of neural and haemal spines in the urostyle in the caudal fin complex were found (Figures 5d,e).

**Figure 6:**
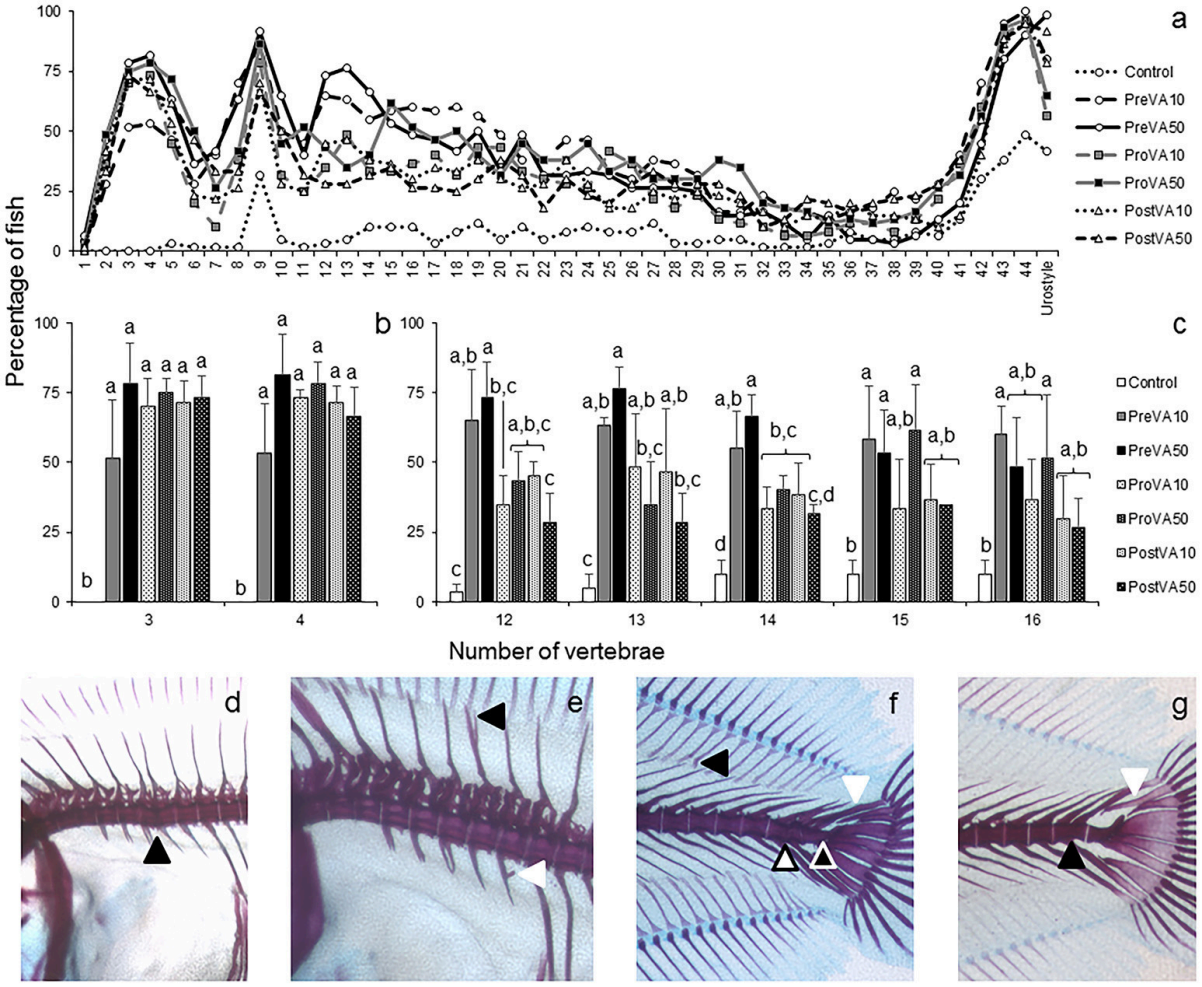
Percentage of Senegalese sole juveniles with a deformity in axial skeleton at 55 dph when fed increased dietary vitamin A levels at different developmental stages. Percentage of juveniles showing a deformity at each vertebra **(a)**, detailed representation of this percentage (mean ± standard deviation) of fish with deformities at the 3rd and 4th vertebrae **(b)**, and at the 12, 13, 14, 15, and 16th vertebrae **(c)**. Detailed view of cephalic vertebrae compression (black arrowhead) **(d)**, miss-joint of neural spines (black arrowhead) and ectopic calcification in a parapophysis (white arrowhead) **(e)**, curved pterigophore (black arrowhead), neural spine-like at the urostyle (white arrowhead), fused preurals (white-filled arrowhead) and straight urostyle (black-filled arrowhead) **(f)**, extra neural spine in preural 1 (black arrowhead) and curved modified neural spine (white arrowhead) **(g)**, in fish at 55 dph. For a detailed description about the different experimental groups, please see the legend of Figure 1. Different letters at the top of each bar denote statistically significant differences among experimental groups (ANOVA, *P* < 0.05; *N* = 3).

When examining skeletal phenotype in soles aged 55 dph, no significant differences in the upper jaw were identified among experimental groups. In contrast, high levels of skeletal deformities were found along the axial skeleton (Figure 6). The Control group showed the lowest percentage of deformed fish at any vertebra considered along the axial skeleton (Figure 6a). Significant differences in the percentage of fish with deformed vertebrae number 3, 4, 12, 13, 14, 15, and 16 were found between Control group and experimental dietary VA unbalanced groups (Figures 6b,c; ANOVA, *P* < 0.05). The typology of deformities was diverse and might be summarized as follows: compressed vertebrae (Figure 6d), neural spine and parapophysis abnormalities (Figure 6e), curved pterigophores, fused vertebrae, straight urostyle, presence of neural spine-like structures at the urostyle (Figure 6f), and supplementary neural spine or curved epural (Figure 6g).

### Thyroidal follicles development

**Figure 7:**
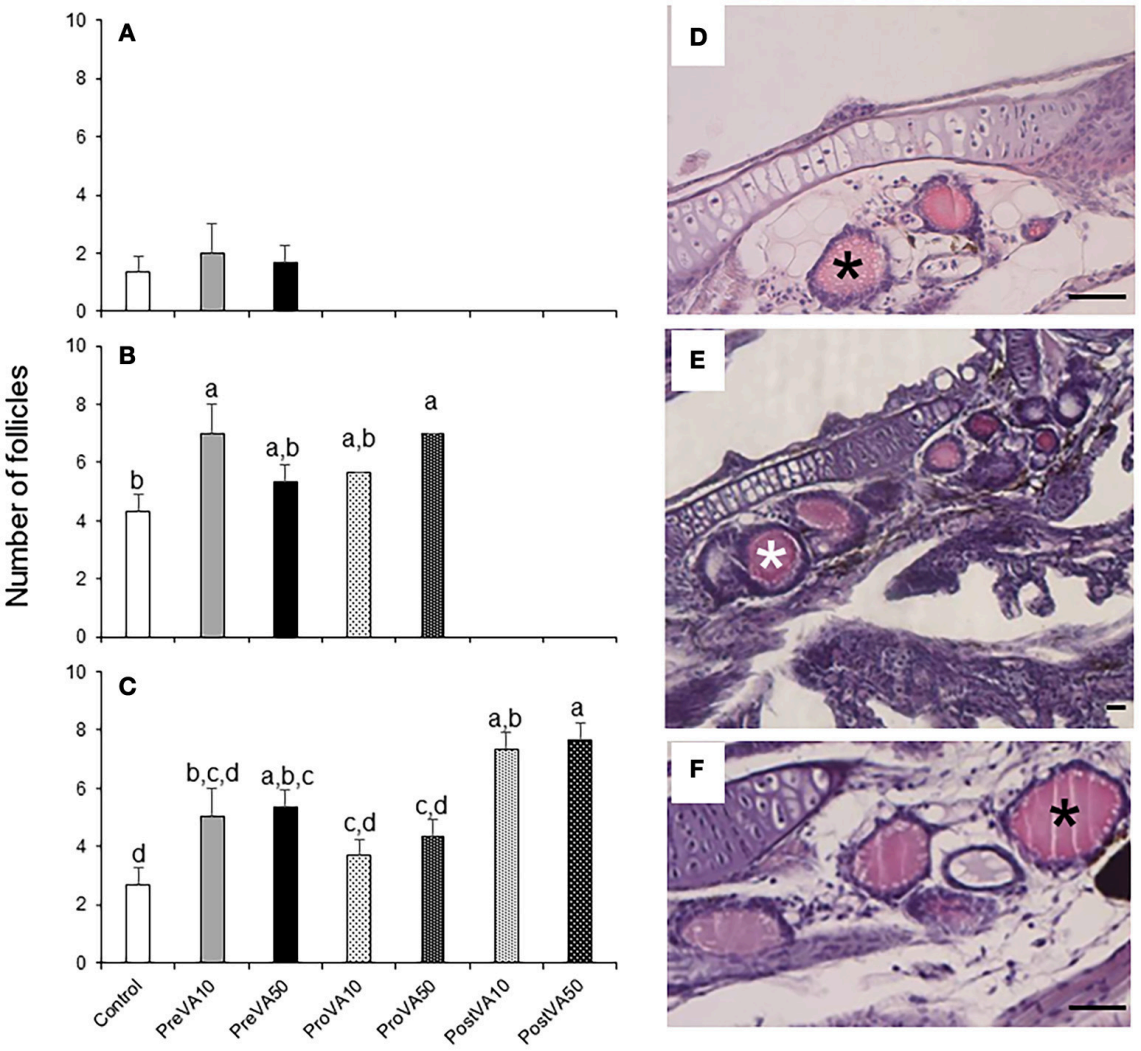
Number of follicles of the thyroid gland (mean ± standard deviation) in Senegalese sole fed with experimental diets at 10 **(A)**, 21 **(B)**, and 55 **(C)** dph. A detailed view of the number of thyroid follicles (black or white asterisks) in a Control **(D)**, PreVA10 **(E)**, and PreVA50 **(F)** larvae at 21 dph. For a detailed description about the different experimental groups, please see the legend of Figure 1. Different letters at the top of each bar denotes statistically significant differences among experimental groups (ANOVA, *P* < 0.05; *N* = 3). Scale bar = 200 μm.

The numbers of follicles appeared similar between the different dietary groups at 10 dph [average values ranging from 2.0 to 1.3 follicles per fish; Figure 7A; ANOVA, *P* > 0.05]. In contrast, specimens from the PreVA10 and ProVA50 groups showed increased number of thyroidal follicles per fish (7.0 ± 1.0) with respect to the Control group (4.3 ± 0.6) at 21 dph (Figure 7B) and similar results were observed at 55 dph (Figure 7C). In contrast, the thyroidal follicle size was not significantly affected by the dietary VA regime in any of the developmental stages (ANOVA, *P* > 0.05; Figures 7D–F).

**Figure 8:**
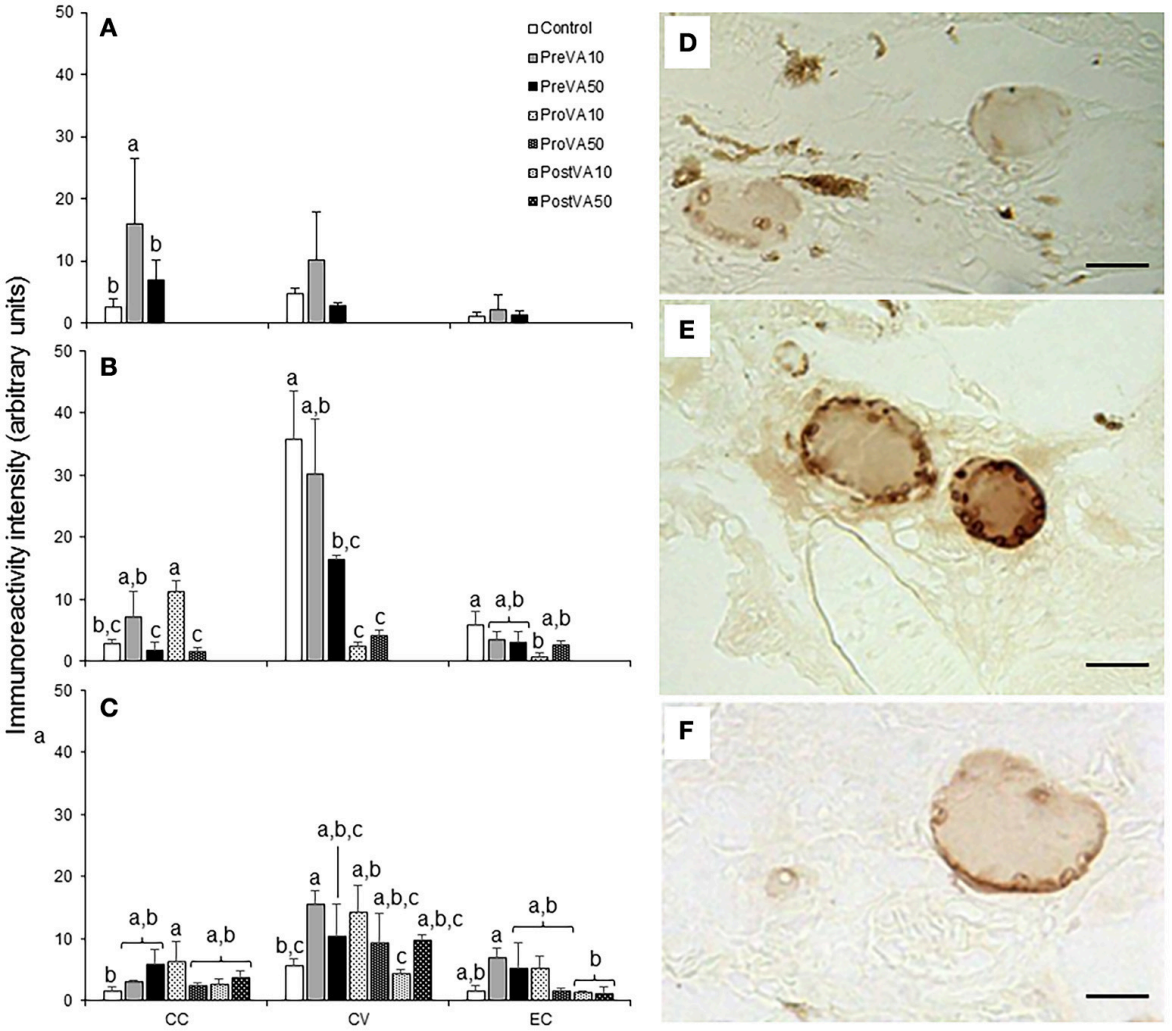
Semi-quantitative assessment of triiodothyronine (T3) hormone (mean ± standard deviation) content by using immunohistochemical approaches in Senegalese sole fed with experimental diets. T3 content at 10 **(A)**, 21 **(B)**, and 55 **(C)** dph. A detailed view of the immunoreactive intensity in thyroid follicles in a Control **(D)**, PreVA10 **(E)**, and PreVA50 **(F)** larvae at 10 dph. *CC*, colloid content; *CV*, cortical vesicles content; *EC*, epithelial cells content. For a detailed description about the different experimental groups, please see the legend of Figure 1. Different letters at the top of each bar denotes statistically significant differences among experimental groups (ANOVA, *P* < 0.05; *N* = 3). Scale bar = 200 μm.

**Figure 9:**
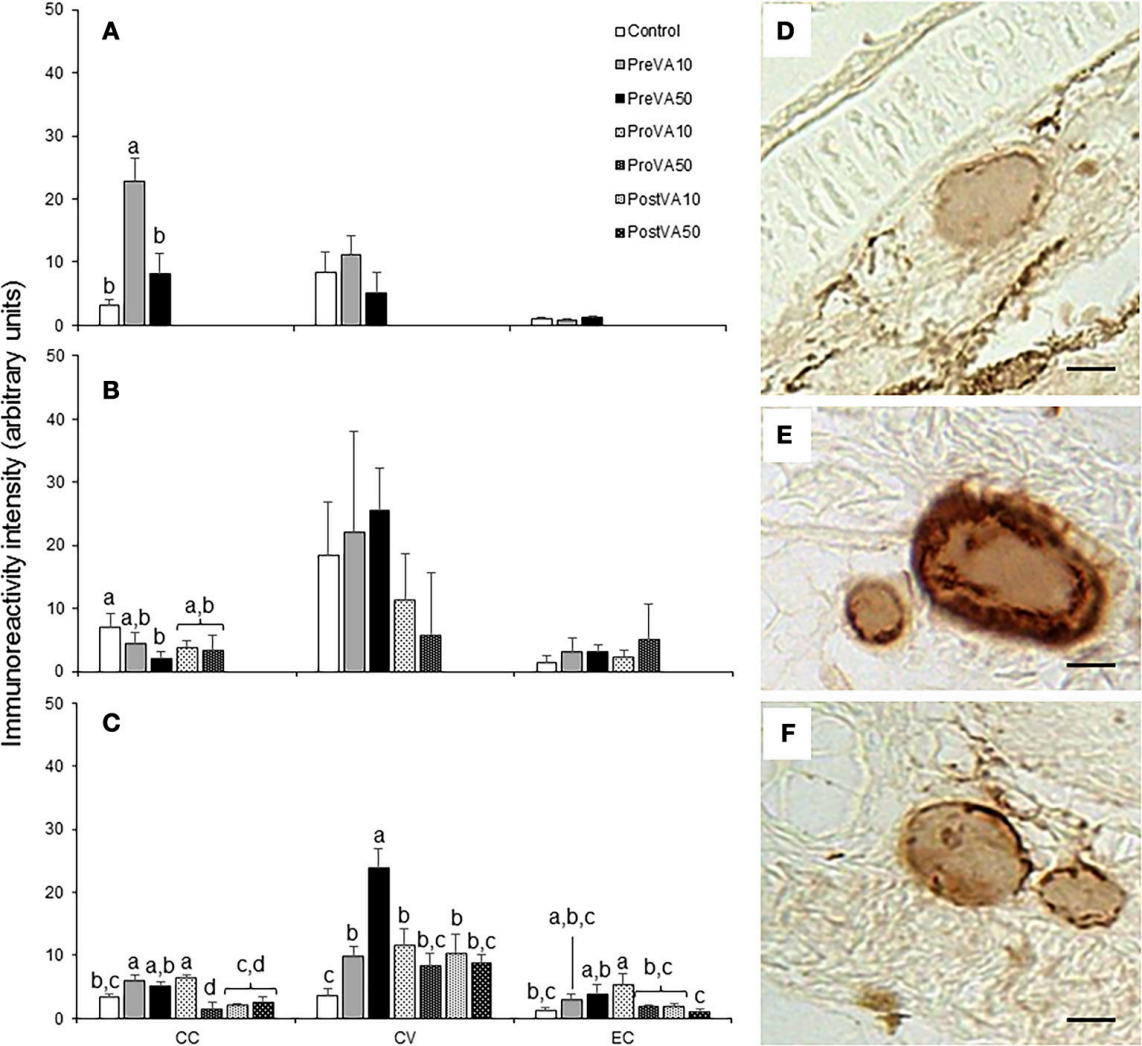
Semi-quantitative assessment of thyroxine (T4) hormone (mean ± standard deviation) content by using immunohistochemical approaches in Senegalese sole fed with experimental diets. T4 content at 10 **(A)**, 21 **(B)**, and 55 **(C)** dph. A detailed view of the immunoreactive intensity in thyroid follicles in a Control **(D)**, PreVA10 **(E)**, and PreVA50 **(F)** larvae at 10 dph. *CC*, colloid content; *CV*, cortical vesicles content; *EC*, epithelial cells content. For a detailed description about the different experimental groups, please see the legend of Figure 1. Different letters at the top of each bar denotes statistically significant differences among experimental groups (ANOVA, *P* < 0.05; *N* = 3). Scale bar = 200 μm.

Semi-quantification results of T3 and T4 at 10, 21, and 55 dph are presented in Figures 8, 9. Fish aged 10 dph from the PreVA10 group showed a higher T3 and T4 immunoreactivity in the colloid content (CC; 16.06 ± 10.54 arbitrary units (AU) and 22.72 ± 3.7 AU, respectively) of the follicles than the rest of the groups (ranging from 2.61 ± 1.26 to 7.0 ± 3.25 AU and from 3.17 ± 0.89 to 8.11 ± 3.38 AU, respectively; ANOVA, *P* < 0.05; Figures 8A, 9A). However, those differences were not found in the cortical vesicles (CV) and epithelial cells (EC) (ANOVA, *P* > 0.05).

At 21 dph, T3 immunoreactivity in CC, CV, and EC were significantly different among experimental groups (Figure 8B; ANOVA, *P* < 0.05). In CC, soles from the PreVA10 and ProVA10 groups exhibited the highest T3 immunoreactivity. However, the higher effect on T3 immunoreactivity has been found in CV with higher signal in the Control and PreVA10 groups (35.78 ± 7.72 and 30.26 ± 8.81 AU; respectively) and lower in ProVA10 and ProVA50 groups (2.51 ± 0.48 and 4.23 ± 0.8 AU, respectively). Regarding T4 immunoreactivity, it was only significantly different in CC, with the Control fish being those showing the highest T4 immunoreactivity (6.92 ± 2.27 AU) and PreVA50 specimens the lowest (1.96 ± 1.22 AU; Figure 9B; ANOVA, *P* < 0.05).

At 55 dph, although significant differences in T3 and T4 immunoreactivity among experimental groups was observed in CC, CV and EC (*P* < 0.05); the highest effect of dietary VA regimens were at the T3 and T4 immunoreactivity in CV (Figures 8C, 9C). In this sense, while PreVA10 specimens have the highest immunoreactivity for T3 (15.49 ± 2.17 AU) and a higher T4 (6.07 ± 0.79 AU) than that of the Control group, it was the PreVA50 group that had the highest T4 immunoreactivity and Control fish the lowest (23.9 ± 3.01 and 3.58 ± 1.19 AU, respectively) in CV (*P* < 0.05). While differences in T3 immunoreactivity at the thyroid follicle's compartments were exemplified in Figures 8D–F; their relatives for T4 immunoreactivity were exemplified in Figures 9D–F.

### Gene expression analyses

**Figure 10:**
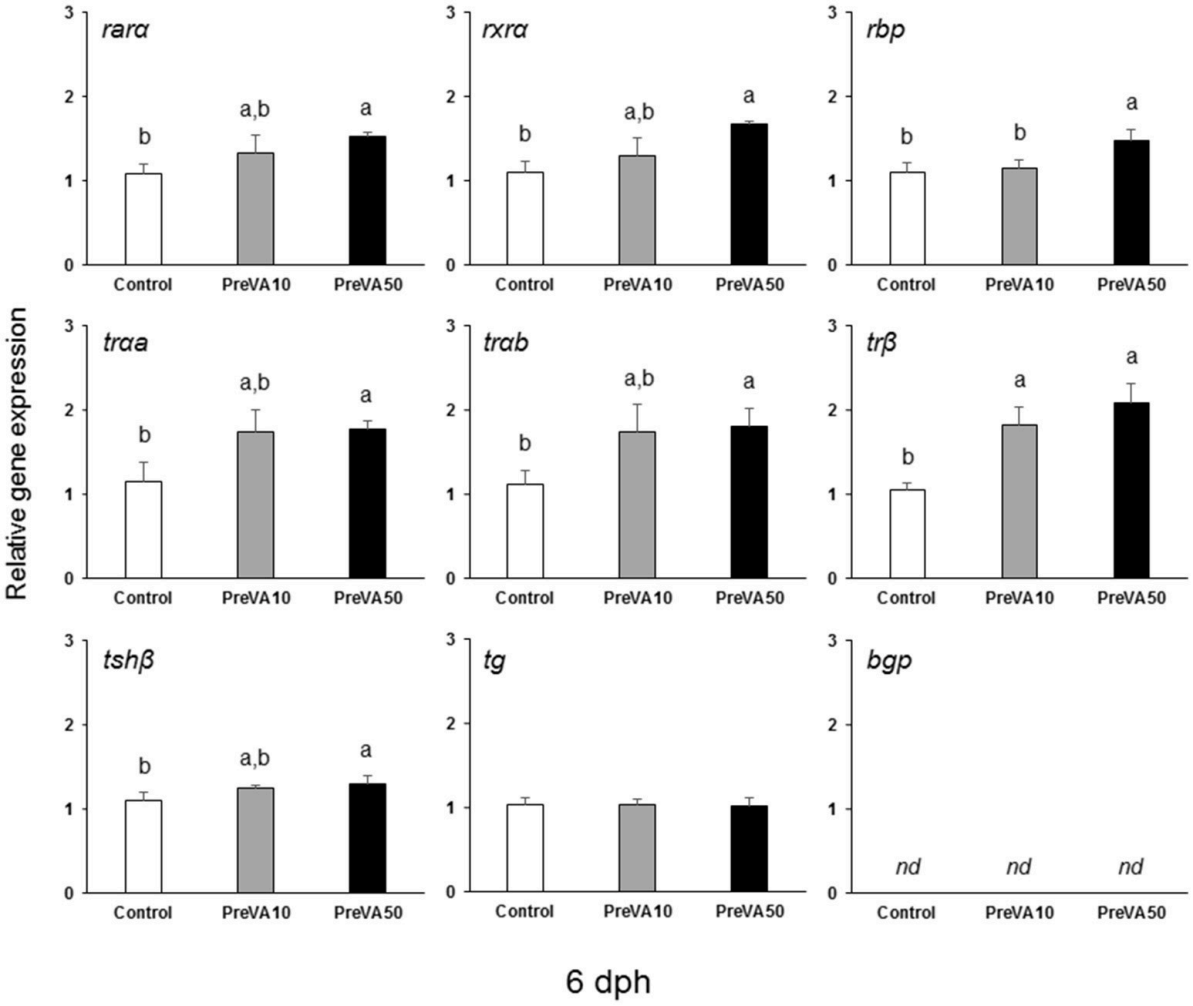
Expression of selected genes related with vitamin A, thyroid hormone and extracellular matrix mineralization in Senegalese sole larvae at 6 dph when fed with increased dietary vitamin A levels during pre-metamorphosis. *rar*α*, retinoic acid receptor* α*; rxr*α*, retinoid x receptor* α*; rbp, retinol binding protein; tr*α*a, thyroid hormone receptor* α *a; tr*α*b, thyroid hormone receptor* α *b; tr*β*, thyroid hormone receptor* β*; tsh*β*, thyroid stimulating hormone* β*; tg, thyroglobulin; bgp, bone gla protein*. For a detailed description about the different experimental groups, please see the legend of Figure 1. Different letters at the top of each bar denotes statistically significant differences among experimental groups (ANOVA, *P* < 0.05; *N* = 3).

**Figure 11:**
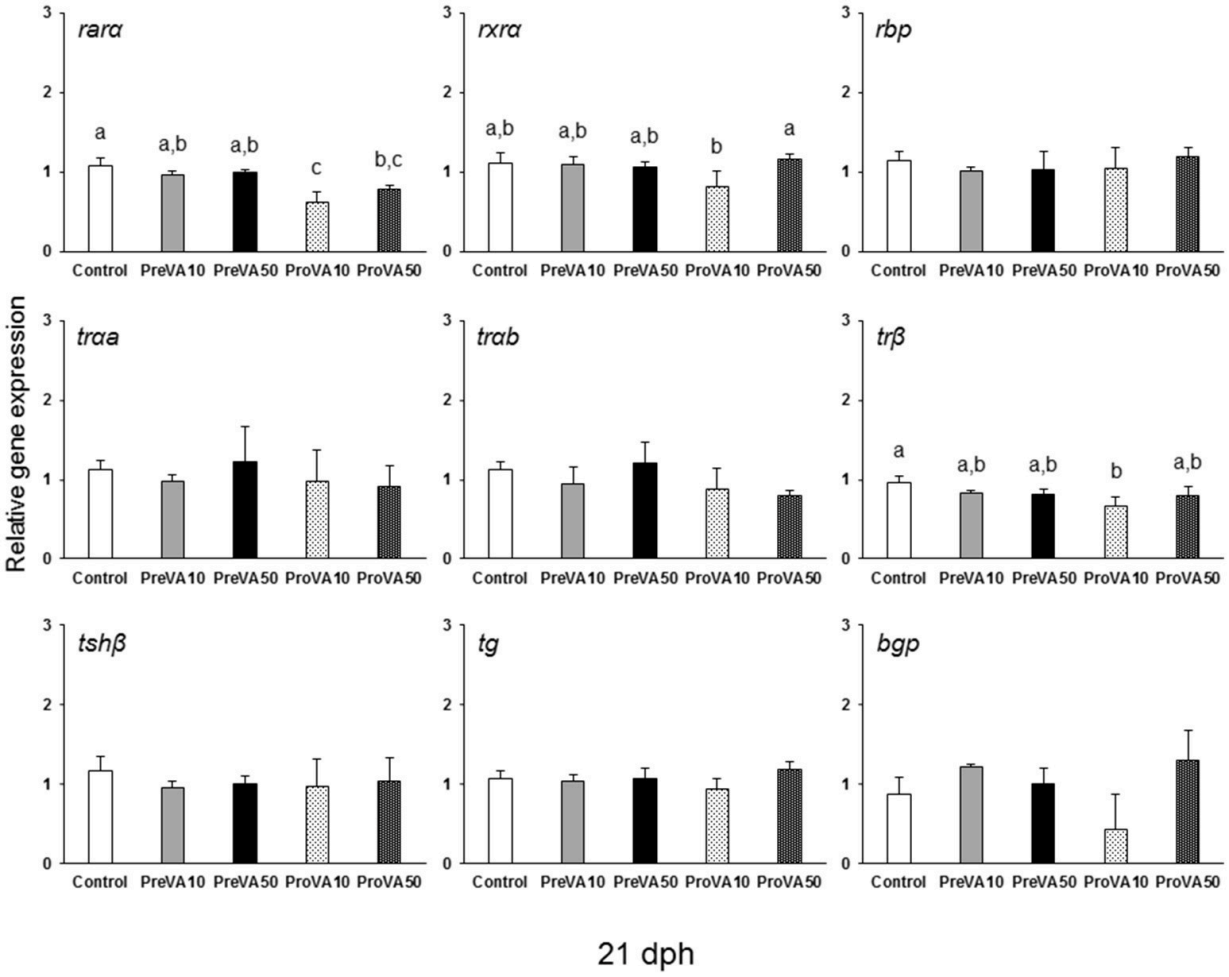
Expression of selected genes related with vitamin A, thyroid hormone and extracellular matrix mineralization in Senegalese sole larvae at 21 dph when fed with increased dietary vitamin A levels during pre- or pro-metamorphosis. *rar*α*, retinoic acid receptor* α*; rxr*α*, retinoid x receptor* α*; rbp, retinol binding protein; tr*α*a, thyroid hormone receptor* α *a; tr*α*b, thyroid hormone receptor* α *b; tr*β*, thyroid hormone receptor* β*; tsh*β*, thyroid stimulating hormone* β*; tg, thyroglobulin; bgp, bone gla protein*. For a detailed description about the different experimental groups, please see the legend of Figure 1. Different letters at the top of each bar denotes statistically significant differences among experimental groups (ANOVA, *P* < 0.05; *N* = 3).

The mRNA levels of *retinoid receptors* (*rar*α and *rxr*α), the *retinol binding protein* (*rbp*), the *th receptors tr*α*a, tr*α*b*, and *tr*β as well as the one regarding the *tsh*β were up-regulated in PreVA50 larvae compared to Control larvae at 6 dph (Figure 10; ANOVA, *P* < 0.05). The expression levels of these genes in PreVA10 larvae was intermediate with respect the PreVA50 and Control groups, with the exception of *tr*β and *rbp*. In particular, while *tr*β was upregulated in PreVA10 larvae with respect to Control group; *rbp* in PreVA10 larvae was significantly lower than that of PreVA50 larvae. At 10 dph, *bgp* expression was still not detected and no statistically significant differences were found in all evaluated genes (ANOVA, *P* > 0.05; results not shown). In contrast, at 21 dph (Figure 11), ProVA10 and ProVA50 larvae showed decreased expression of *rar*α with respect to the Control group, while PreVA10 and PreVA50 specimens intermediate values (ANOVA, *P* < 0.05). In contrast, *rxr*α expression was only different between the ProVA50 and ProVA10 groups (ANOVA, *P* < 0.05). The *tr*β appeared significantly down-regulated in ProVA10 soles compared to the Control group (ANOVA, *P* < 0.05). Finally, at 41 dph, no differences were detected in all evaluated genes (ANOVA, *P* > 0.05; results not shown).

## Discussion

Several studies have evaluated the effects of dietary VA imbalances at early development in different marine fish species (Fernández and Gisbert, 2011; Negm et al., 2013, 2014; Lie et al., 2016). A dietary safe level for VA during larval development was initially suggested to be less than 50,000 IU Kg^−1^ (Takeuchi et al., 1995); although (Mazurais et al., 2009) showed that the optimal level for harmonious skeletal development significantly fluctuated during ontogeny of fish. Furthermore, in our previous works it has been shown how dietary VA excess affected skeletal development, the number and size of thyroid follicles, TH biosynthesis (Fernández et al., 2009); endochondral bone ossification were more prone to develop abnormalities than intramembranous bone ossification (Fernández and Gisbert, 2010); and the need for integrative research on fish nutritional requirements, since the same dietary VA content that hampered skeletal development stimulated fish immunocompetence (Fernández et al., 2015). The present work investigated by means of biochemical, phenotypic, histological, immunohistochemical and transcriptional approaches, how dietary VA excess influenced fish morphogenesis in Senegalese sole when it was applied at particular early developmental stages (pre-, pro- and post-metamorphosis).

### Retinoid content in live prey and sole specimens

At a commercial level, feeding early life stages of most marine fish species is still relying on live prey like rotifers and *Artemia*, which nutritional value should be improved with enriching emulsions (Hamre et al., 2013). The nutritional value/composition of live feeds and their enriching emulsions varies greatly. In particular, the VA level in different commercial enriching emulsions may vary up to 700 times (from 2117.2 IU Kg^−1^ in Aquagrow® DHA to 1.5^*^10^6^ IU Kg^−1^ in Easy Selco®). Furthermore, previous research studies showed that copepods (natural preys of marine fish larvae) do not contain the same amounts and VA sources (as well as other nutrients) as those found in commercially enriched rotifers and *Artemia* (Ronnestad et al., 1998; van der Meeren et al., 2008). Thus, regardless of the difficulties in enriching live preys such as rotifers and *Artemia* metanauplii with the proper VA levels (Giménez et al., 2007), two different levels of VA representative of the differences found in commercial enriching emulsions (above mentioned) were successfully incorporated in live preys by means of retinyl palmitate supplementation. Levels of retinoic acid (RA), retinol, retinyl palmitate and total VA content in live preys were in line with previous studies when emulsions were supplemented with increasing levels of VA (Giménez et al., 2007; Negm et al., 2013, 2014; Lie et al., 2016).

The content of retinoids in Senegalese sole at the end of each developmental stage (pre-, pro-, and post-metamorphosis) clearly reflected that of enriched live prey. Furthermore, no RA or retinal was detected regardless the feeding regime, in agreement with Fernández et al. (2009), and confirming the proper management of dietary retinoids by Senegalese soles (Boglino et al., 2017). Retinol content in each experimental group at the different sampling points vaguely reflected the level of dietary VA administered at different developmental stages, and only significant differences were found between soles at post-metamorphosis when fed the highest dietary VA content during this developmental stage (PostVA50 group). Furthermore, after the VA-washing period, no differences in retinol content were found among experimental groups, suggesting that Senegalese sole juveniles efficiently metabolize this retinoid compound, a key regulatory step to avoiding undesired effects on retinoic signaling pathways (Boglino et al., 2017). Unlike with retinol content, retinyl palmitate levels and total VA content were more consistent with the hypervitaminosis A induced by the diets at the different developmental periods: the higher VA content in live feed led to higher retinyl palmitate (and total VA) content in Senegalese sole individuals. Surprisingly, even after the VA washing period, specimens fed with highest dietary VA content during post-metamorphosis (PostVA50) still presented higher retinyl palmitate levels than the Control group, suggesting that VA washing period was not long enough to return the retinyl palmitate body content to normal levels. The increasing accumulation of retinyl palmitate concomitant with a decrease in retinol levels during development was in agreement with previous studies (Moren et al., 2004; Fernández et al., 2009).

### Senegalese sole performance

Dietary VA imbalance has already been demonstrated to affect larval growth performance and survival in different fish species (Fernández and Gisbert, 2011; Negm et al., 2013, 2014; Lie et al., 2016). However, those effects have been found to be species- (Fernández, 2011), developmental- (Villeneuve et al., 2006), and cell type-dependent (Fernández et al., 2014). In the present study, no differences in SL were found, in contrast with results from Fernández et al. (2009) where a lower dietary excess (up to 8 times higher than the control diet) provided during a longer time (from 6 to 37 dph) induced lower SL values. These results suggest that even low dietary imbalances are able to affect growth in SL when administered during longer periods (whole metamorphosis process). In contrast, while a temporally defined VA imbalance (particularly during pre-metamorphosis stage; present work) affected DW, it did not when lower dietary imbalances were supplied during longer periods (Fernández et al., 2009). Regarding Senegalese sole morphology, body width was shown to be more critically time-dependent, since early juveniles (55 dph) exposed to dietary VA imbalance at pre-metamorphosis were more round-shaped than those challenged during pro- and post-metamorphosis. In line with these observations, altered body morphology was also reported in European seabass subjected dietary VA excess during larval development (Georga et al., 2011).

Curiously, an acceleration of the eye migration process was previously observed in Senegalese sole exposed to dietary VA excess during pre-metamorphosis (Fernández et al., 2009). However, even though the dietary VA excess used in the present study was higher than in the previous one, its supply during pre- and pro-metamorphosis did not disrupt nor accelerate the eye migration process, suggesting that the exposure time (between 6 and 10 dph in Fernández et al. (2009) and between 3 and 6 dph [rotifer feeding phase] in the present study) was more likely responsible for such differences. Altogether, these results suggest that early morphogenesis is differentially affected depending on the magnitude of VA imbalance and the time frame during which it is supplied and thus, dietary VA content is of the utmost importance for harmonic early vertebrate development to obtain a normal post-metamorphic phenotype.

### Skeletal development under dietary VA imbalance at different early developmental phases

Pre-metamorphic sole larvae fed with increased levels of VA exhibited higher frequency of skeletal deformities in the axial skeleton and higher mean number of vertebral bodies; the last presumably as a consequence of a disruption in notochord segmentation (Haga et al., 2009). Present results are in agreement with the notion of the earlier the VA imbalance the higher the effect (Haga et al., 2002; Villeneuve et al., 2006). Most importantly, a different degree of mineralization in particular structures was also observed depending on the dietary VA dose supplied and the developmental period considered. For instance, while at 10 dph PreVA10 larvae had a higher degree of mineralization of upper and lower jaws, haemal and neural spines compared to Control larvae, larvae in the PreVA50 group at 10 dph did not. *In vivo* and *in vitro* works have already suggested VA accelerates skeletal mineralization (Fernández et al., 2011, 2014; Cardeira et al., 2016). Surprisingly, while a non-dose response on this process was observed when larvae were fed with the highest VA content (PreVA50); a dose-response on the increased mineralization degree was observed when dietary regimens were supplied during pro-metamorphosis. Skeletal structures undergoing mineralization from 10 to 21 dph (hyoid, interhyal, pre-opercular, quadrate, those composing the splanocranium, and haemal and neural spines) showed a higher mineralization stage at 21 dph in ProVA10 and ProVA50 than in Control group. In contrast, no differences in degree of mineralization in any skeletal structure considered was observed regardless the level of dietary VA supplied during post-metamorphosis, suggesting that metamorphosed Senegalese sole larvae have an increasing capacity to control VA metabolism. Overall this suggested that although skeletogenesis was disrupted in soles fed dietary VA supplemented diets, mineralization degree seemed to be more dependent on the dietary VA regime supplied at different metamorphic stages.

### Thyroid follicles development and THs biosynthesis/compartmentalization are disrupted differentially

A previous study demonstrated that dietary VA excess provided from 6 to 37 dph increased the number and size of thyroid follicles, as well as the T3 and T4 total immunoreactivity (Fernández et al., 2009). In contrast, when an excess of dietary VA was provided during a limited time period (present study), only an increased number of follicles was found, regardless of the metamorphosis period considered (pre-, pro-, and post-metamorphosis). Here, in addition, we observed a differential immunoreactivity effect for both THs depending on the thyroid follicle's compartment considered and the developmental stage in which the VA excess was supplied. In this sense, during pre-metamorphosis, the higher effect of a VA imbalance was on THs biosynthesis (increasing colloid content of THs); while during pro-metamorphosis, the higher effect of a VA imbalance seemed to be on TH's mobilization (decreasing THs in cortical vesicles, more evidenced in T3). Furthermore, previous disruption (particularly during pre-metamorphosis) in development of thyroid follicles and THs homeostasis was still reflected in metamorphosed fish, indicating a higher sensitivity when the surge of THs appear to trigger the metamorphosis process (Manchado et al., 2008b). Curiously, during this particular metamorphic stage, a similar pattern of degree of mineralization at 10 dph (increased mineralization in PreVA10 specimens, but similar to that of Control group in PreVA50 specimens) was found for T3 and T4 levels (PreVA10 with increased immunoreactivity and PreVA50 with a similar one to the Control group); evidencing a point where the VA dose-response in TH immunoreactivity was also broken and might be responsible for the skeletal phenotype observed in the PreVA10 group.

### Gene expression of VA and TH signaling pathways

The retinoic acid (RAR), retinoid X (RXR) and thyroid (TRs) receptors control vertebrate development and homeostasis through genomic and non-genomic actions (Ross et al., 2000; Balmer and Blomhoff, 2002; Gogakos et al., 2010). Interestingly, RXRα represents a crosstalk point between both signaling pathways since it forms heterodimers with either RARs or TRs, thus controlling gene transcription (Gogakos et al., 2010). More recently, it has been suggested that, through the control of histone modifications, an elevated TH signaling might stimulate RA signaling genes, while RA signaling might de-repress TH signaling (Li et al., 2015). In fish, metamorphosis is a largely known THs signaling driven process (Campinho et al., 2010; Gomes et al., 2015); while RA and THs signaling pathways crosstalk in flatfish metamorphosis (eye migration and adult pigmentation acquisition) has been recently hypothesized (Shao et al., 2016). Nevertheless, although transcriptomic approaches have been previously used to uncover the underlying VA signaling pathways activated or inhibited in fish species (Oliveira et al., 2013; Lie et al., 2016), little is known about the interaction between RA and TH signaling pathways determining the skeletal development in fish.

In general, the present study showed as the expression of most analyzed genes was significantly altered at 6 dph under dietary VA excess, while a lower proportion showed an altered expression at 21 dph, or showed no modification at 41 dph. These results were in line with the increasing effect of dietary VA imbalance supplied at each time period on skeletogenesis, follicle development and THs immunoreactivity: the earlier the imbalance, the higher the effect. In this sense, a disrupted expression of VA receptors (*rara* and *rxra*) in a dietary VA dose-dependent manner has been reported at 6 dph, while at 21 dph only the expression of *rara* was also consistently perturbed under a dietary VA imbalance. These results are in line with our previous *in vivo* and *in vitro* reports where the isoform *rara* has been shown the most consistently disrupted VA receptor under dietary VA excess in fish species (Fernández et al., 2011, 2014). However, although an RA exposure led also to an upregulation of *rara* expression, it was concomitant with a down-regulation of *tr*α*a* and *tr*β*1* expression in another flatfish species (Shao et al., 2016). Here we observed that although increased dietary VA levels at 6 dph increased *rara* expression, it also led to an upregulation of *tr*α*a, tr*α*b*, and *tr*β. Such contradictory results might be due to species-specific ontogenetic differences (metamorphosis starts at 210°C days and ends at 525°C days in Japanese flounder, while metamorphosis in Senegalese sole begins and ends at 170 and 340°C days, respectively), and/or with the experimental approach (RA bath exposure vs. dietary VA imbalance, respectively). Furthermore, such differences seem to be correlated with the different effects observed on the eye migration process during pre-metamorphosis, being inhibited in RA exposed Japanese flounder, but not in Senegalese sole fed high dietary VA content.

In agreement with the previous species-specific differences in metabolism and response to dietary VA imbalances, here we reported an increased gene expression of *rbp* only at 6 dph in PreVA50 larvae (fed with 30 times more the total VA content than the Control group), while in gilthead sea bream larvae *rbp* was found up-regulated when dietary VA content was increased 1.5 times, but down-regulated when it was increased 10 times (Fernández et al., 2011). Since RBP is the main protein responsible of retinol transport (Funkenstein, 2001), such differences reinforce this notion of the nutritional requirements and sensitivity to dietary VA content being fish species-specific.

Similarly to different vertebrate species, two isoforms of TRs (TRα and TRβ) have been found in several fish species, although others like the Senegalese sole had genes encoding further isoforms like *tr*α*a* and *tr*α*b* (Manchado et al., 2008b; Pittman et al., 2013). All isoforms were differentially expressed in pre-metamorphic larvae fed increasing levels of VA (6 dph), while only the expression of the *tr*β isoform was still altered at pro-metamorphosis (21 dph). Furthermore, *tr*β was equally up-regulated in soles fed both supplemented dietary VA levels, while *tr*α*a* and *tr*α*b* showed intermediate expression values between those of the Control and PreVA50. Several reports indicated that both TR isoforms may play an important role in bone morphogenesis, being expressed at both intramembranous and endochondral bone formation sites (reviewed in Basset and Williams, 2016). However, since present results were obtained from a whole body RNA extraction, and these receptors are ubiquitously expressed in Senegalese sole tissues (Manchado et al., 2008b); we cannot relate the altered gene expression of each TR with a specific tissue, and particularly to that of bone tissue. Nevertheless, taking into account the loss-of-function approaches in mice (reviewed in Basset and Williams, 2016), a higher gene expression of *tr*α*a* and *tr*α*b* in larvae fed increased VA doses at 6 dph should present an advanced mineralization at the end of pre-metamorphosis, which only occurred in PreVA10 larvae (the ones showing intermediate values). Following the same line of argument, high *tr*β expression in soles fed increased dietary VA contents at 6 (PreVA10 and PreVA50) and 21 dph (ProVA10 and ProVA50) should induce a lower degree of mineralization in skeletal structures. However, since TRs function on skeletogensis is more dependent on the presence of their ligand (T3), these unexpected phenotypes might be better explained by the observed disruption on THs biosynthesis/compartmentalization. In this sense, the Senegalese sole skeletal phenotype was in accordance with the pattern of TH immunoreactivity: the higher the T3 immunoreactivity the higher degree of mineralization in skeletal structures. Such correlation was clear at 6 dph, larvae from PreVA10 group that had increased T3 immunoreactivity and a higher degree of mineralization at the end of pre-metamorphosis than those from the Control and PreVA50 groups. Similarly, the highest degree of mineralization of soles fed increased VA levels during pro- and post-metamorphosis might be correlated with the highest number (although with equal size) of thyroid follicles in those specimens at 21 and 55 dph, respectively.

Levels of THs are regulated through the pituitary–thyroid axis mainly by thyroid stimulating hormone (TSH) and thyroglobulin (TG), among other factors (Pittman et al., 2013). Their role on TH synthesis in the thyroid follicle and its negative regulation by TH are well established in Senegalese sole (Manchado et al., 2008a). In Senegalese sole, *tsh*β expression decreased from 3 dph to the start of metamorphosis (Manchado et al., 2008a), only being up-regulated in the present study at 6 dph in PreVA50 larvae. In contrast, *tg* expression has been shown to increase sharply at the onset of metamorphosis, but decrease after the climax of metamorphosis (Manchado et al., 2008a). Since TG is known to act as a matrix for thyroid hormone biosynthesis, its impairment has been associated with abnormal TH biosynthesis (Targovnik et al., 2011). Thus, the present unaltered *tg* expression at any sampling time, regardless of the developmental period and the dietary VA level supplied, might be interpreted as Senegalese sole from all experimental groups having normal TH biosynthesis, this being in agreement with the reported lack of effect on the eye migration process. Altogether, considering results at the transcriptional level, histological T3 and T4 immunoreactivity and the increased number of follicles, the lack of effect on eye migration process and the increased mineralization of particular structures, the differential effects of applying a dietary VA imbalance at clearly distinct metamorphic stages seems to be due (at least in part) to an altered THs release from thyroid follicles and abnormal TH signaling in targeted tissues, rather than abnormal biosynthesis of THs.

Finally, among the known ECM mineralization gene markers, the most largely used are *alkaline phosphatase* and *bone gla protein* (*bgp*, also known as *osteocalcin*). Among the several different alkaline phosphastase isoenzymes, the *non-tissue specific alkaline phosphatase* (expressed in bone, kidney and liver) is regularly assessed for bone ECM mineralization (Le Du and Millán, 2002). In contrast, although a broad range of whole-organism physiological roles have been recently attributed to *bgp* (Karsenty and Ferron, 2012) it has been extensively described as a specific bone marker (Pinto et al., 2001). Thus, since total RNA extraction was performed in whole body animals, *bgp* expression evaluation might be a more accurate assessment of ECM mineralization. Expression of *bgp* has been not detected at 6 and 10 dph (pre-metamorphosis), while an increasing expression was found at 21 and 41 dph. Such ontogenetic-related expression was in line with its expression being directly related to the calcification of skeletal structures (Gavaia et al., 2006). Nevertheless, regardless of the level of dietary VA and the developmental period considered, its expression remained unaffected. Contradictory results on the effect of VA imbalance were previously reported. While an up-regulation of *bgp* was found *in vivo* and *in vitro* in gilthead sea bream (Fernández et al., 2011, 2014) under hypervitaminosis A and under exposure to RA in an *ex vivo* approach with Atlantic cod (*Gadus morhua*; Lie and Moren, 2012), a down-regulation *in vivo* was reported in Atlantic cod fed increasing levels of VA (Lie et al., 2016). Our lack of *bgp* expression disruption in Senegalese sole larvae could be due to its low expression level (Cts > 30) in sole larvae, not allowing an accurate detection of differences among experimental groups. Such low expression seems to be related with the lower degree of mineralization of skeletal structures in Senegalese sole, a passive swimming fish, as sustained swimming increased the mineral content of fish vertebra (Totland et al., 2011).

Altogether, present results showed a lack of dose-response effect on mineralization degree due to dietary VA levels supplied, but following the same trend shown for THs semiquantification at thyroid follicles: the highest mineralization the highest THs immunoreactiveness. This might be due to differences on VA metabolism under different dietary VA contents at each developmental window, as particularly seen at pre-metamorphosis where the VA dose-response in bone mineralization (and THs immunoreactiveness) was specifically broken. At this developmental time, *rbp* expression was significantly higher in VA50 fish group than in VA10 and Control groups, thus suggesting that a specific VA metabolism process has been triggered. Further and specific research work on this issue might shed some light on how VA metabolism is modulated by dietary VA content since different reports showed or not a clear modulation on the expression of genes involved in VA metabolism (Oliveira et al., 2013; Lie et al., 2016; Boglino et al., 2017).

## Conclusions

Although the impact of dietary VA is well known in vertebrates, the signaling pathways involved remain to be properly understood. The present study evidenced that the dietary VA level provided to Senegalese sole at different developmental stages (pre-, pro-, and post-metamorphosis) had a differential impact: the earlier the nutritional imbalance applied, the higher the effect on skeletogenesis. Furthermore, VA effects seemed to be, at least partially, due to impaired THs metabolism and signaling as shown by the perturbed development of thyroid follicles, THs compartmentalization in thyroid follicles and whole body expression of *trs*. Our results suggest an interaction between both signaling pathways (VA and TH) to be dose and developmental time dependent and highlight the suitability of Senegalese sole as a model species for developmental biology studies on how specifically TH and VA interact during early vertebrate development.

## Author contributions

Conceived and designed the experiments: IF and EG. Performed the experiments: IF and EG. Analyzed the data: IF, JO, and FH. Wrote the paper: IF, JO, MD, FH, KA, MM, CS, and EG.

### Conflict of interest statement

The authors declare that the research was conducted in the absence of any commercial or financial relationships that could be construed as a potential conflict of interest. The reviewer LS and handling Editor declared their shared affiliation, and the handling Editor states that the process nevertheless met the standards of a fair and objective review.
