# Erratum: Vitamin A Affects Flatfish Development in a Thyroid Hormone Signaling and Metamorphic Stage Dependent Manner

**DOI:** 10.3389/fphys.2017.00647

**Published:** 2017-08-29

**Authors:** Frontiers Production Office

**Affiliations:** Frontiers Media SA Lausanne, Switzerland

**Keywords:** vitamin A, thyroidal follicles, skeletogenesis, ossification, nuclear receptors, flatfish, Senegalese sole, *Solea senegalensis*

Due to a production error, the Institute in Franciso Hontoria's affiliation (affiliation 4) was incorrectly given as “Instituto de Ciencias Marinas de Andalucía (CSIC).” The correct affiliation is: “Instituto de Acuicultura de Torre de la Sal (CSIC), Torre de la Sal, Castellón, Spain”. The publisher apologizes for this mistake. The original version of this article has been updated.

