# Supplementary material for: Vitamin A Affects Flatfish Development in a Thyroid Hormone Signaling and Metamorphic Stage Dependent Manner
Source: Front Physiol. 2017 Jun 30;8:458. doi: 10.3389/fphys.2017.00458 (PMC5492123; doi:10.3389/fphys.2017.00458)
Supplement: Supplementary file 1 [file Table1.DOCX]

Supplementary Material

**Vitamin A Affects Flatfish Development in a Thyroid Hormone Signaling and Metamorphic Stage Dependent Manner**

Ignacio Fernández*, Juan B. Ortiz-Delgado, Maria J. Darias, Francisco Hontoria, Karl B. Andree, Manuel Manchado, Carmen Sarasquete, and Enric Gisbert

*** Correspondence:** Ignacio Fernández, *Centro de Ciências do Mar (CCMAR), Universidade do Algarve, Campus de Gambelas, 8005-139 Faro (Portugal)*. Tel.: +351 289800057; E-mail: [nacfm@hotmail.com](mailto:nacfm@hotmail.com); [ivmonzon@ualg.pt](mailto:ivmonzon@ualg.pt); Web address: <http://www.bioskel.ccmar.ualg.pt/>

# Supplementary Tables

**Supplementary Table 1.** Gene name, accession numbers (GenBank), primers and Taqman® probes used for relative quantification of gene expression in Senegalese sole (*Solea senegalensis*) under dietary vitamin A nutritional imbalance at different developmental stages.

| **Gene name - *abbreviation*** | **Accession number*** | | **Component** | **5’ to 3’ nucleotide sequences** | **E**** | **Expected amplicon size (bp)** |
| --- | --- | --- | --- | --- | --- | --- |
| *ubiquitin - ubq* | | AB291588 | Forward | GCCCAGAAATATAACTGCGACAAG | 1.93 | 70 |
|  |  |  | Reverse | TGACAGCACGTGGATGCA |  |  |
|  |  |  | Probe | ACTTGCGGCATATCAT |  |  |
| *retinoic acid receptor alpha - rarα* | | AB668026 | Forward | GAAGAAGAAGGACGAGAAGAAGCA | 1.97 | 77 |
|  |  |  | Reverse | TGTCTATCATCTGCTCCGTGTCT |  |  |
|  |  |  | Probe | CAGGACGTAGCTCTCC |  |  |
| *retinoic X receptor alpha - rxrα* | | AB668024 | Forward | CTCATCGTTCCATAGCCGTTAAAGA | 2.11 | 68 |
|  |  |  | Reverse | GCTGTTGCGGTGAACGT |  |  |
|  |  |  | Probe | TCGCCAACAGAATCC |  |  |
| *thyroid hormone receptor alpha a - trαA* | | AB366000 | Forward | CCGCCTCATTGTCCTGTGA | 1.93 | 61 |
|  |  |  | Reverse | GGACATTGGCTCGGTTTAACCT |  |  |
|  |  |  | Probe | TTGGCCGCTGGACCAC |  |  |
| *thyroid hormone receptor alpha b - trαB* | | AB444623 | Forward | GAAGCTGGTGCTAAACGGTAGAT | 1.87 | 63 |
|  |  |  | Reverse | CCTCCATCCTTCCCCTACAAAA |  |  |
|  |  |  | Probe | ACGATGGCCCTTCCCC |  |  |
| *thyroid hormone receptor beta - trβ* | | AB366001 | Forward | CAGAAGCGGAAGTTCCTGAGT | 1.89 | 96 |
|  |  |  | Reverse | TTTGTTTCCTTCAGGTGTGTTTGC |  |  |
|  |  |  | Probe | ACGCATGACCAATATC |  |  |
| *thyriod stimulating hormone beta - tshb* | | AB297482 | Forward | GAACCAGTGCGGACAGAGTA | 1.89 | 60 |
|  |  |  | Reverse | CAGGAAAAGGGTAGATACGTGTGA |  |  |
|  |  |  | Probe | AAGTGCAGCAAACCAG |  |  |
| *thyroglobulin -tg* | | AB297481 | Forward | GACCGCCGCCTCTCT | 1.96 | 52 |
|  |  |  | Reverse | TCCTGACGAAGCTGGACATG |  |  |
|  |  |  | Probe | CTCGCCGTGATGACCT |  |  |
| *retinol binding protein -rbp* | | FF290795 | Forward | CCAGTAAGCAGATCTCCCTCTTCT | 1.89 | 76 |
|  |  |  | Reverse | TCCCGTCATATCACTGGTCTGA |  |  |
|  |  |  | Probe | CCATCGCCTGGTCCTC |  |  |
| *bone Gla protein - bgp* | | AY823525 | Forward | TCGCTGCCTACACCACCTA | 2.15 | 60 |
|  |  |  | Reverse | GATGAACAACGGTTTGGTGCTAAAA |  |  |
|  |  |  | Probe | CTATGGACCAATTCCC |  |  |

*GeneBank

**Efficiency (2=100%)
