# Supplementary material for: Vitamin A Affects Flatfish Development in a Thyroid Hormone Signaling and Metamorphic Stage Dependent Manner
Source: Front Physiol. 2017 Jun 30;8:458. doi: 10.3389/fphys.2017.00458 (PMC5492123; doi:10.3389/fphys.2017.00458)
Supplement: Supplementary file 2 [file Image1.PDF]

*Supplementary Material*

# Vitamin A Affects Flatfish Development in a Thyroid Hormone Signaling and Metamorphic Stage Dependent Manner

Ignacio Fernández*, Juan B. Ortiz-Delgado, Maria J. Darias, Francisco Hontoria, Karl B. Andree, Manuel Manchado, Carmen Sarasquete, and Enric Gisbert

*** Correspondence:** Ignacio Fernández, *Centro de Ciências do Mar (CCMAR), Universidade do Algarve, Campus de Gambelas, 8005-139 Faro (Portugal)*. Tel.: +351 289800057; E-mail: nacfm@hotmail.com; ivmonzon@ualg.pt; Web address: http://www.bioskel.ccmar.ualg.pt/

## 1 Supplementary Figures

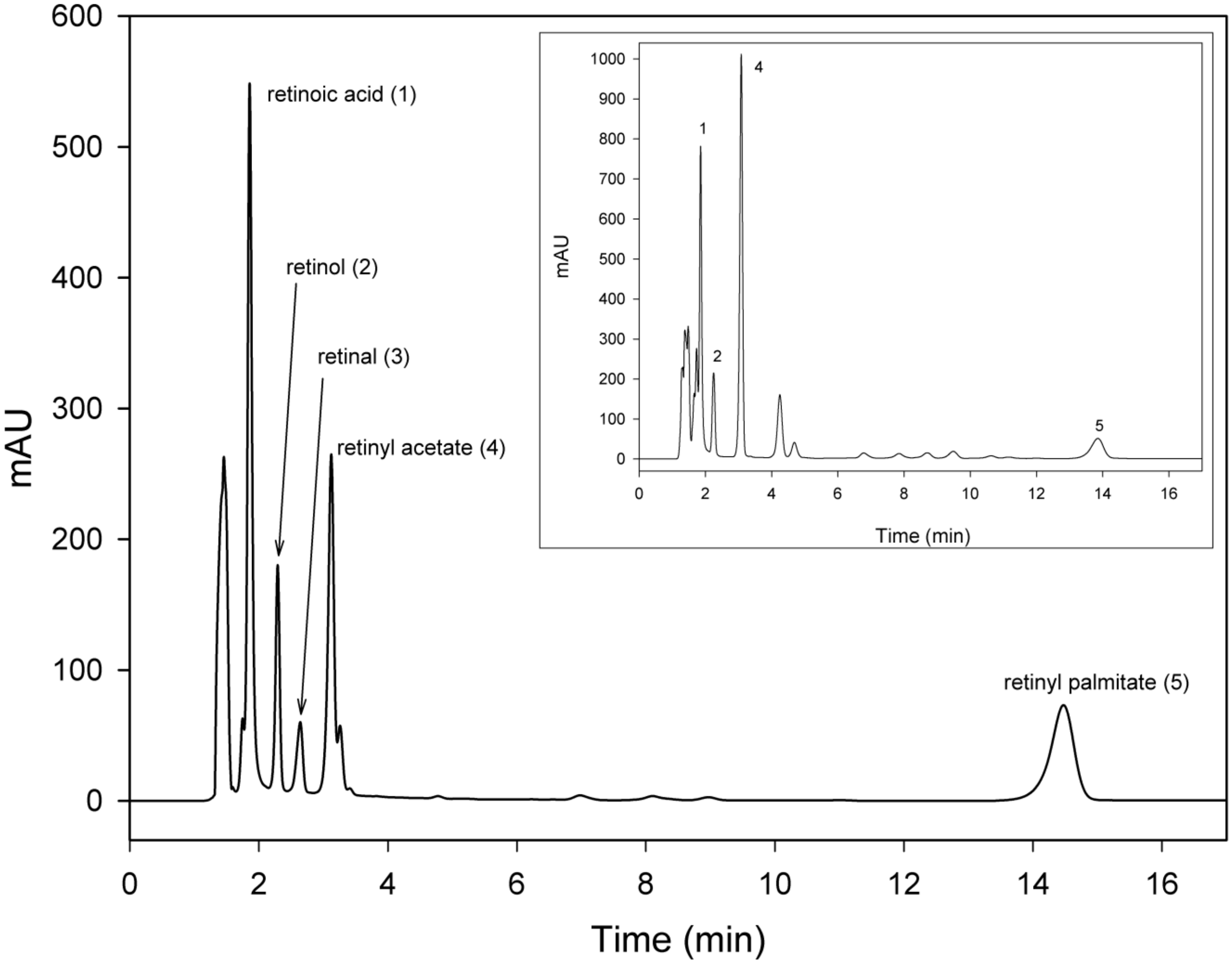


**Supplementary figure 1.** Separation of standard retinoids (retinoic acid, retinol, all-trans retinyl acetate and retinyl palmitate) by HPLC using a reverse phase column Lichrospher C-18 and a UV–visible detector set at 325 nm, with the inner picture showing an example of the correspondent peaks of retinoids from an enriched rotifers sample.
