# Supplementary material for: Vitamin A Affects Flatfish Development in a Thyroid Hormone Signaling and Metamorphic Stage Dependent Manner
Source: Front Physiol. 2017 Jun 30;8:458. doi: 10.3389/fphys.2017.00458 (PMC5492123; doi:10.3389/fphys.2017.00458)
Supplement: Supplementary file 3 [file Image2.PDF]

Supplementary Material

Vitamin A Affects Flatfish Development in a Thyroid Hormone Signaling and Metamorphic Stage Dependent Manner

Ignacio Fernández*, Juan B. Ortiz-Delgado, Maria J. Darias, Francisco Hontoria, Karl B. Andree, Manuel Manchado, Carmen Sarasquete, and Enric Gisbert

*** Correspondence:** Ignacio Fernández, *Centro de Ciências do Mar (CCMAR), Universidade do Algarve, Campus de Gambelas, 8005-139 Faro (Portugal)*. Tel.: +351 289800057; E-mail: nacfm@hotmail.com; ivmonzon@ualg.pt; Web address: http://www.bioskel.ccmar.ualg.pt/

1 Supplementary Figures

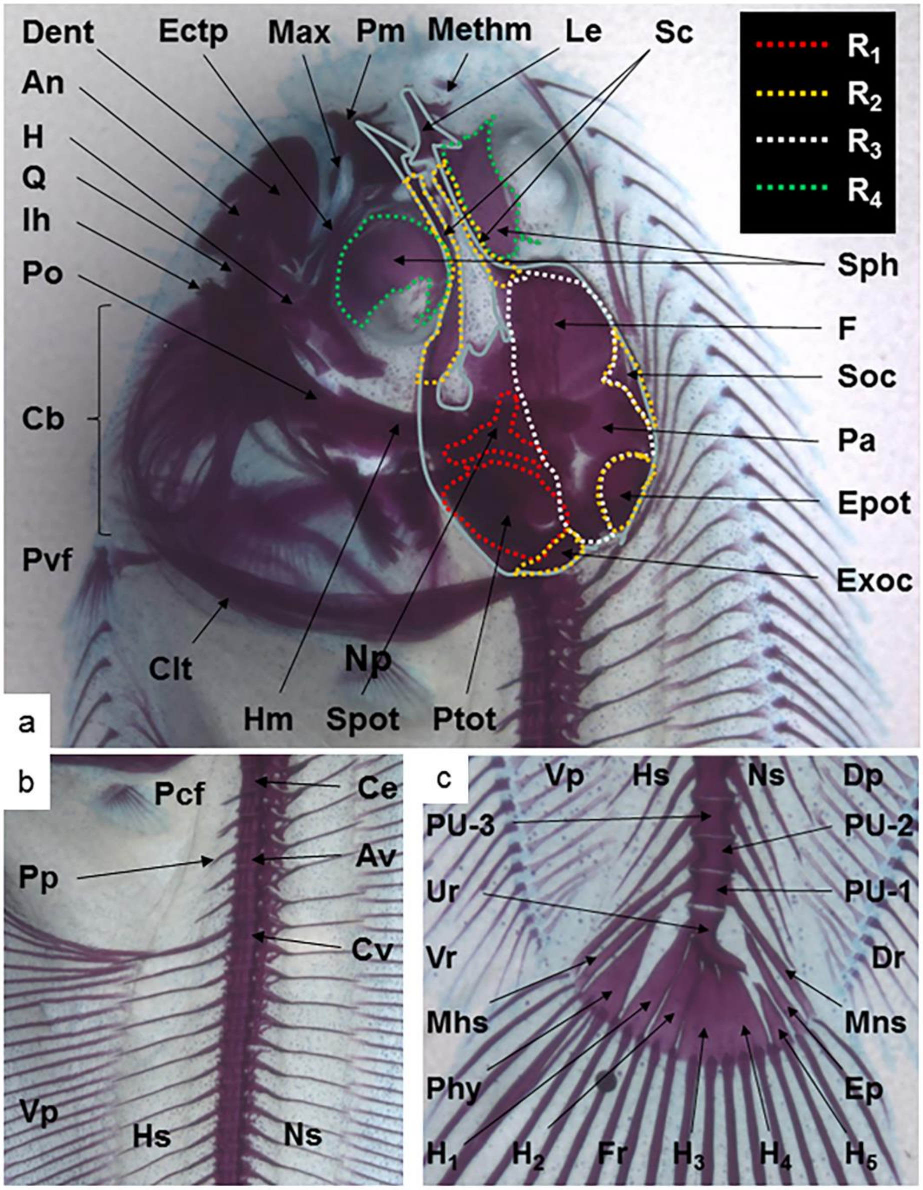

Supplementary figure 2. Cranial and axial regions and skeletal structures in Senegalese sole juveniles to evaluate mineralization degree and skeletogenic quality. Detailed view of cranial (a), axial (b) and caudal (c) structures. *An*, anguloarticular; *Av*, abdominal vertebrae; Cb, ceratobranchials; *Ce*, cephalic vertebrae; *Clt*, cleithrum; *Cv*, caudal vertebrae; *Dent*, dentary; *Dp*, dorsal pterigophores; *Dr*, dorsal rays; *Ectp*, ectopterygoid; *Ep*, epural; *Exoc*, exoccipital; *F*, frontal bone; *Fr*, fin rays; *H*, hyoid; H_1, hypural 1; H_2, hypural 2; H_3, hypural 3; H_4, hypural 4; H_5, hypural 5; *Hm*, hyomandibular; *Hs*, heamal spines; *Ih*, interhyal; *Le*, lateral ethmoid; *Max*, maxillary; *Methm*, mesethmoid; *Mhs*, modified haemal spines; *Mns*, modified neural spine; *Ns*, neural spines; *Pa*, parietal; *Pcf*, pectoral fin; *Phy*, Parahypural; *Pm*, pre-maxila; *Po*, pre-opercular; *Pp*, parapophysis; *Ptot*, pterotic; *PU-1*, preural vertebra 1; *PU-2*, preural vertebra 2; *PU-3*, preural vertebra 3; *Pvf*, pelvic fin; *Q*, quadrate; *Sc*, supraorbital canal bones; *Soc*, supraoccipital; *Sph*, sphenoid; *Spot*, sphenotic; *Ur*, urostyle; *Vp*, ventral pterigophores; *Vr*, ventral rays. In color dotted lines depict the regions considered for evaluating the degree of mineralization in the neurocranium: R_1, region 1; R_2, region 2; R_3, region 3; R_4, region 4.
