# Supplementary material for: Vitamin A Affects Flatfish Development in a Thyroid Hormone Signaling and Metamorphic Stage Dependent Manner
Source: Front Physiol. 2017 Jun 30;8:458. doi: 10.3389/fphys.2017.00458 (PMC5492123; doi:10.3389/fphys.2017.00458)
Supplement: Supplementary file 4 [file Image3.PDF]

Supplementary Material

Vitamin A Affects Flatfish Development in a Thyroid Hormone Signaling and Metamorphic Stage Dependent Manner

Ignacio Fernández*, Juan B. Ortiz-Delgado, Maria J. Darias, Francisco Hontoria, Karl B. Andree, Manuel Manchado, Carmen Sarasquete, and Enric Gisbert

*** Correspondence:** Ignacio Fernández, *Centro de Ciências do Mar (CCMAR), Universidade do Algarve, Campus de Gambelas, 8005-139 Faro (Portugal)*. Tel.: +351 289800057; E-mail: nacfm@hotmail.com; ivmonzon@ualg.pt; Web address: http://www.bioskel.ccmar.ualg.pt/

1 Supplementary Figures

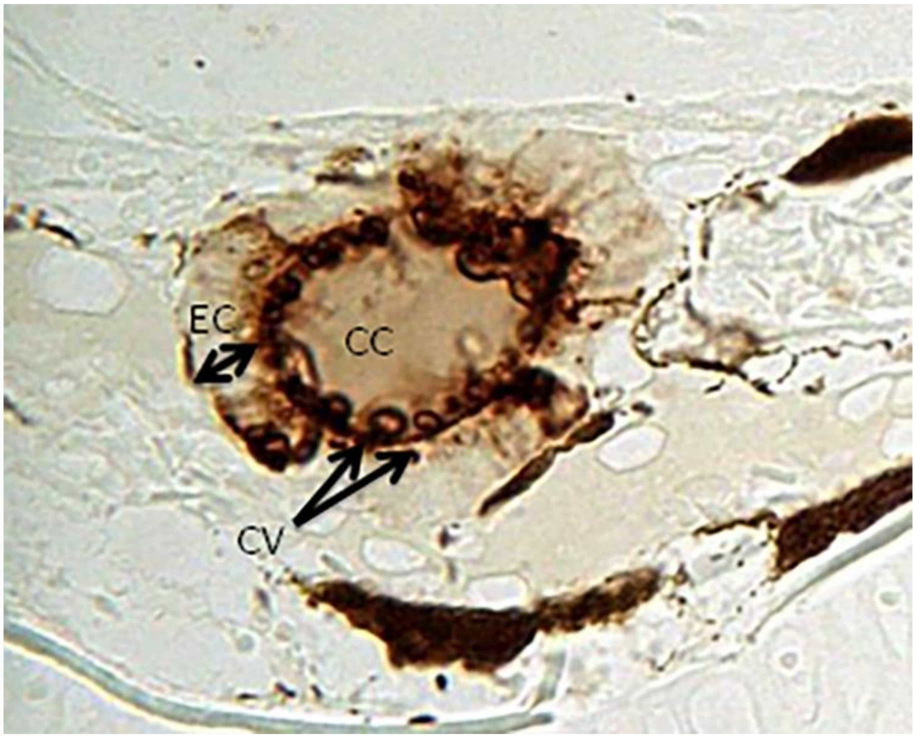

Supplementary figure 3. Detailed view of a thyroid follicle and showing the three different regions/contents considered for the evaluation of thyroid hormone's immunoreactivity. *CC*, colloid content; *CV*, cortical vesicles content; *EC*, epithelial cells content.
