# Supplementary material for: Vitamin A Affects Flatfish Development in a Thyroid Hormone Signaling and Metamorphic Stage Dependent Manner
Source: Front Physiol. 2017 Jun 30;8:458. doi: 10.3389/fphys.2017.00458 (PMC5492123; doi:10.3389/fphys.2017.00458)
Supplement: Supplementary file 5 [file Image4.PDF]

Supplementary Material

Vitamin A Affects Flatfish Development in a Thyroid Hormone Signaling and Metamorphic Stage Dependent Manner

Ignacio Fernández*, Juan B. Ortiz-Delgado, Maria J. Darias, Francisco Hontoria, Karl B. Andree, Manuel Manchado, Carmen Sarasquete, and Enric Gisbert

*** Correspondence:** Ignacio Fernández, *Centro de Ciências do Mar (CCMAR), Universidade do Algarve, Campus de Gambelas, 8005-139 Faro (Portugal)*. Tel.: +351 289800057; E-mail: nacfm@hotmail.com; ivmonzon@ualg.pt; Web address: http://www.bioskel.ccmar.ualg.pt/

1 Supplementary Figures

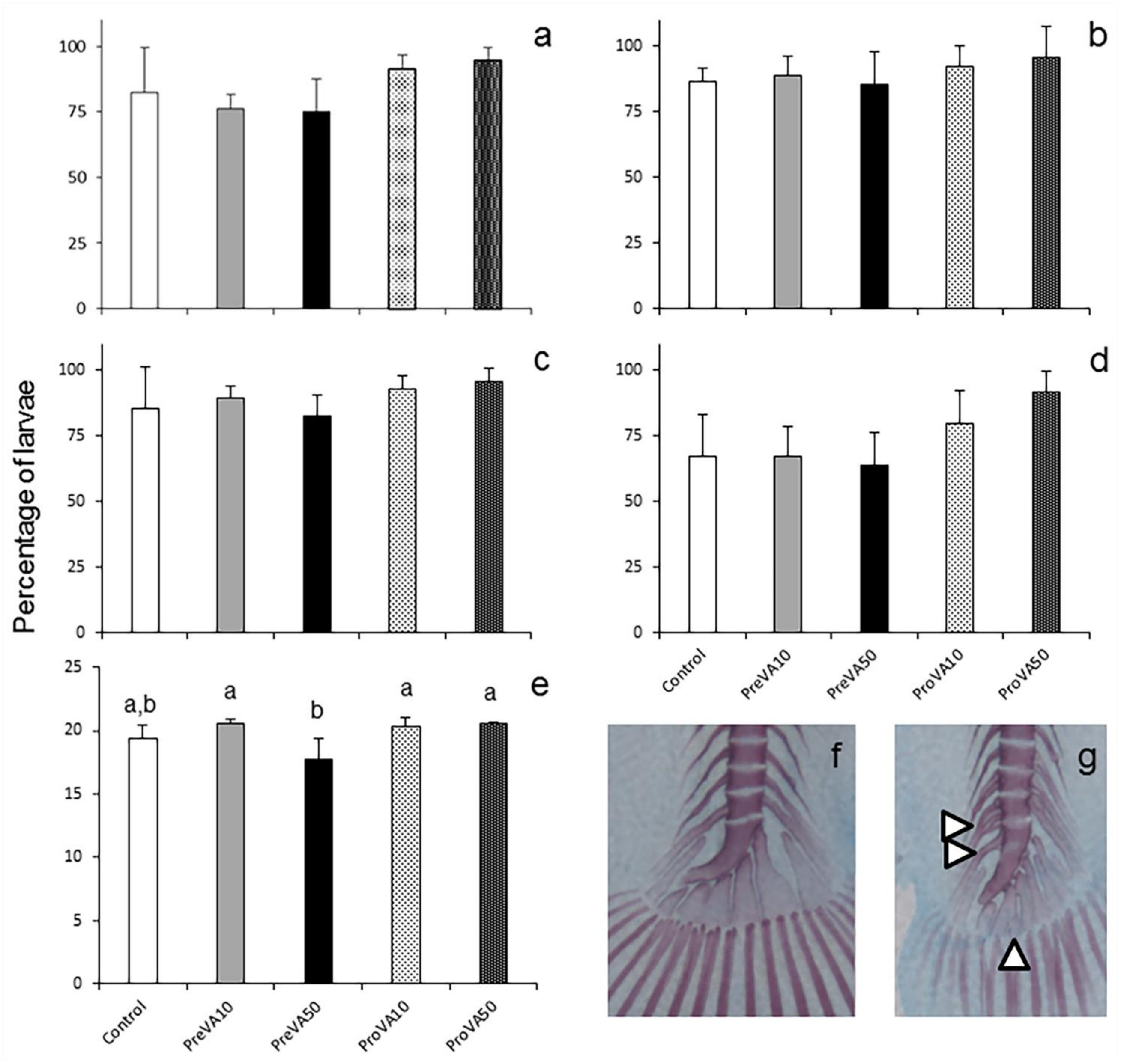

Supplementary figure 4. Mineralization degree of different skeletal structures in 21 dph Senegalese sole larvae fed with increased dietary vitamin A levels at different developmental stages. Percentage of larvae showing mineralized vertebrae (a), Mns (b) hypural 1 (c), hypural 5 (d) and mean number of formed caudal rays (e). A detailed view of the mean number of caudal rays from PreVA10 (f) and PreVA50 (g) larvae at 21 dph is presented. Also note the several caudal deformities (white arrowheads) in the PreVA50 larvae, namely at the neural spine of preural vertebra 1, the modified neural spine and the hypurals morphology. For a detailed description about the different experimental groups, please see the legend of figure 1. Different letters at the top of each bar denotes statistically significant differences among experimental groups (ANOVA, $P < 0.05$; N=3).
